# Randomized clinical trial comparing efficacy and safety of brand versus generic alendronate (Bonmax^®^) for osteoporosis treatment

**DOI:** 10.1371/journal.pone.0180325

**Published:** 2017-07-05

**Authors:** Aasis Unnanuntana, Atthakorn Jarusriwanna, Panupan Songcharoen

**Affiliations:** Department of Orthopaedic Surgery, Faculty of Medicine Siriraj Hospital, Mahidol University, Bangkok, Thailand; Garvan Institute of Medical Research, AUSTRALIA

## Abstract

**Introduction:**

Although the same efficacy and tolerability are anticipated due to both drugs containing the same active ingredients, comparative studies between brand and generic alendronate are limited. Accordingly, the objective of this study was to compare efficacy and safety between brand alendronate and a recently introduced generic alendronate drug.

**Methods:**

A total of 140 postmenopausal women or men aged older than 50 years who met the indications for osteoporosis treatment were randomized to receive either generic (Bonmax^®^) or brand alendronate (Fosamax^®^) 70 mg/week over a 12-month period during the May 2014 to June 2015 study period. Endpoints included bone mineral density (BMD) changes at the lumbar spine, total hip, and femoral neck; percentage of patients with predefined levels of change in total hip and lumbar spine BMD at 12 months; and, changes in biochemical bone markers at 3, 6, and 12 months. Tolerability was evaluated by patient self-reporting of adverse experiences.

**Results:**

At 12 months post-treatment, BMD significantly increased at all sites in both groups. There were no differences in BMD percentage changes or the number of patients with stable or increased BMD after 1 year between groups. No significant differences in the amount of biochemical bone marker reduction or incidence of adverse events were observed between groups.

**Conclusions:**

Generic and brand alendronate produced similar gains in BMD and reduction in bone turnover markers. Both medicadoitions were also equally well-tolerated. Based on these findings, generic alendronate (Bonmax^®^) is a viable alternative to the original brand of alendronate.

**Trial registration:**

ClinicalTrials.gov NCT02371252

## Introduction

Osteoporosis is a systemic disease characterized by compromised bone strength that predisposes individuals to increased risk of fracture [1]. Fragility fracture is associated with premature mortality [2] and substantial decrease in quality of life (QOL) [3]. Moreover, both short- and long-term care for patients with fragility fractures place an enormous economic burden on the health care system [4]. Due to high personal and societal costs of fragility fracture, prevention is critically important. Once an individual has been identified as being at high risk for fracture, such as those diagnosed with osteoporosis or those with a history of fragility fracture, an appropriate pharmacological intervention should be employed.

According to the 2010 Thai osteoporosis guideline, bisphosphonate is considered as a first-line therapy [5]. Bisphosphonates have been shown to significantly reduce the risk of both vertebral and non-vertebral (including hip) fractures by reducing bone turnover and increasing bone mass, thus improving bone strength [6]. Alendronate, which is available in a once-weekly formulation, is one of the most commonly used oral bisphosphonates, and it has been approved for the treatment and prevention of postmenopausal osteoporosis [7]. Previous studies have shown continued efficacy and safety of alendronate after 10 years of continued use [8,9].

Due to the escalating cost of health care, governments in many countries have instituted numerous measures to minimize health care-related expenditures. One of the most commonly used cost-saving methods is to encourage physicians to use generic substitution for brand drugs after the patents on these brand drugs have expired [10–12]. Alendronate is the first amino-bisphosphonate to lose patent protection, thus allowing generic duplication of this drug. Insurance entities and group health care providers prefer physicians to prescribe generic alendronate instead of the brand drug due to its lower cost. Although it is expected that generic alendronate will have the same clinical efficacy as the brand formulation based on bioequivalence data, clinical information regarding side effects and the effect of generic alendronate on bone mineral density (BMD) and fracture reduction is limited. Accordingly, the objective of this study was to compare efficacy and safety between brand alendronate and a recently introduced generic alendronate drug. The use of generic alendronate could be recommended if adequate efficacy is established, and if it has the same safety profile as brand alendronate.

## Materials and methods

This study was a 12-month randomized controlled, non-inferiority trial conducted at Siriraj Hospital, Mahidol University. The protocol and consent forms used were approved by the Siriraj Institutional Review Board (SIRB), Faculty of Medicine Siriraj Hospital, Mahidol University on 22 April 2014. This study was registered at ClinicalTrials.gov via the Protocol Registration and Results System (PRS) (NCT02371252) on 7 September 2014. The 4–5 month delay in protocol registration was due to time needed for translation of the protocol from Thai language to English language (S1 and S2 Files). A detailed informed consent form was signed by each participating patient, and all patient information was kept confidential. The study design and reporting format were based on CONSORT (Consolidated Standards of Reporting Trials) principles (S1 Checklist). The authors confirm that all ongoing and related trials for this drug/intervention are registered.

### Participants

All patients were recruited from the metabolic bone disease clinic of Siriraj Hospital. Included participants were community-dwelling, ambulatory men or postmenopausal women (defined as no vaginal bleeding or spotting for at least 12 months) >50 years who met the indications for osteoporosis treatment. According to the Thai Osteoporosis Foundation 2010 treatment guideline for osteoporosis [5], pharmacological treatment should be considered in postmenopausal women and men ≥50 years that have one of the following indications: previous history of a low-energy hip or vertebral compression (clinical or morphometric) fracture; having a T-score at the lumbar spine, femoral neck, or total hip ≤-2.5; having a low BMD (T-score at the lumbar spine, femoral neck, or total hip between -1.0 and -2.5) with a 10-year probability of hip fracture ≥3% or a 10-year probability of a major osteoporosis-related fracture ≥20% according to FRAX^®^, the World Health Organization’s fracture risk assessment tool [13,14].

We prospectively screened and recruited patients who had indications for osteoporosis treatment during May 2014 to June 2015. Since we prospectively collected data of each patient from baseline to 12-months post-treatment, follow-up data of all patients were obtained during August 2014 to June 2016. Patients with any one of the following conditions were excluded: history of severe dyspepsia or gastroesophageal reflux disease; presence of hypocalcemia (serum calcium <8.5 mg/dL), severe vitamin D deficiency (serum 25-hydroxyvitamin D <10 ng/mL), or metabolic bone diseases other than postmenopausal osteoporosis; presence of estimated glomerular filtration rate (eGFR) <35 mL/min/1.73 m^2^; history of any bisphosphonate or anabolic agent use within the past year; or, history of glucocorticoid use (≥5 mg/day of prednisolone or its equivalent) within the past 6 months.

Patients who met the inclusion criteria were enrolled and sequentially assigned an allocation number (allocation ratio 1:1) into either the generic alendronate group or the brand alendronate group. The randomization sequence was concealed prior to enrollment. Although patients were not blinded to the given medication, the physicians and the research assistant who collected the data were blinded to each patient’s assigned study medication. Patient group allocation was determined using a computer-generated blocked randomization scheme, using block sizes of two and four. Patients in the generic alendronate group received Bonmax^®^ (Apotex Incorporated, Toronto, Ontario, Canada) 70 mg/week for 12 months, whereas patients in the brand alendronate group received Fosamax^®^ (Merck Sharp & Dohme (Italia) S.P.A., Pavia, Italy) 70 mg/week for 12 months. The study medication was taken with a full of glass of water (6–8 oz) upon waking after an overnight fast. After taking the medication, patients were instructed to remain in an upright position for at least 30 minutes before eating their first meal of the day. In addition to the study medication, calcium and vitamin D supplementation were given to all patients in both groups.

Study visits were planned at baseline, and at 3, 6, and 12 months after treatment. At each visit, blood samples were collected and study medication was provided. Residual unused medication supplied at the previous visit was collected and counted. A trained research assistant evaluated possible side effects and adverse events. Worsening of a pre-existing medical condition and/or occurrence of a new fracture were considered adverse events. If a new hip fracture occurred during the study period, the medication was stopped due to concern that bisphosphonate may interfere with the fracture healing process [15].

### Assessments

#### Bone mineral density testing

BMD was measured by Dual Energy X-ray Absorptiometry (DXA) at the lumbar spine and proximal femur at baseline and 12 months after treatment. BMD was measured on the same Lunar densitometer instrument (GE Healthcare, Little Chalfont, United Kingdom) at both visits. An independent investigator who was blinded to the treatment allocation performed the BMD analyses. Change in BMD from baseline to 12-month post-treatment was classified into three types: increased, stable, and decreased BMD. A gain or loss of BMD beyond the *in vivo* least significant change was defined as increased or decreased BMD, respectively. The least significant change in BMD was calculated as 2.8 × coefficient of variation. Since the coefficient of variation for lumbar spine measurements in clinical practice is usually approximately 1%, a change of ≥3% in lumbar spine BMD was considered to be significant; however, a change at the proximal femur was not significant until it was ≥5% (the coefficient of variation for proximal femur measurement was 1.8) [16]. Changes in BMD within 3% at the lumbar spine or within 5% at the proximal femur were considered to be stable BMD.

#### Biochemical assessment

Differences in efficacy of generic and brand alendronate were also evaluated by determining the response of bone turnover markers. We used serum β-isomerized C-terminal telopeptides (β-CTx; Roche Diagnostics Elecsys, Mannheim, Germany) and serum total procollagen type 1 amino-terminal propeptide (P1NP; Roche Diagnostics Elecsys, Mannheim, Germany) to assess the bone turnover rate. Serum β-CTx was used to monitor the bone resorption rate, while serum P1NP was used to monitor the rate of bone formation. All blood samples were drawn at approximately 8 am after 12 hours of fasting. These samples were analyzed at the central laboratory at our hospital. Laboratory data were collected at baseline, and at 3, 6, and 12months after bisphosphonate treatment. Basic laboratory tests were also performed at each visit, including serum total calcium, albumin, phosphate, parathyroid hormone, blood urea nitrogen, creatinine, alkaline phosphatase, and 25-hydroxyvitamin D (25(OH)D).

#### QOL assessment

We used the European Quality of Life Scale (EuroQol) to assess QOL of patients in both groups. The EuroQol instrument is designed for self-completion by the participant and it contains two parts: the EQ-5D-5L utility score (EQ-US) and the EQ visual analogue scale (EQ-VAS). In this study, we used only the EQ-VAS. Patients were asked to score their health status on a visual analog scale (VAS) that ranged from 0 to 100. The top score of the scale (100) represents the best imaginable health state, with 0 representing the worst imaginable health status [17,18].

#### Efficacy and safety determinations

We determined the efficacy of both generic and brand alendronate by evaluating the mean percentage change in BMD from baseline to 1-year post-treatment. The primary outcome of this study was the mean percentage change in lumbar spine BMD from baseline to 1-year post-treatment. We chose BMD at the lumbar spine as our primary outcome because of the rapid and large gains in BMD observed at this site in response to bisphosphonate treatment [19–21]. Secondary BMD endpoints included mean percentage change from baseline to 1-year post-treatment in BMD of the femoral neck and total hip. Percentage changes in biochemical bone markers from baseline to 3-, 6-, and 12-months post-treatment were included as secondary efficacy endpoints. Safety was monitored during the study by recording clinical and laboratory adverse events. Patients were encouraged to report any potential adverse events to the investigators at any time during the study period.

### Statistical analyses

In order to test non-inferiority between generic and brand alendronate groups, we used standard deviation of BMD change at the lumbar spine from baseline to 1-year post-treatment for brand alendronate (Fosamax^®^). A previous investigation [10] found that the standard deviation of BMD at the lumbar spine in patients who received brand alendronate to be 0.138. Based on the results of that study, we set a non-inferiority margin between brand and generic alendronate equal to half of the standard deviation of the brand alendronate group. Power analysis and sample size calculations indicated that a sample size of 50 patients per group would provide 80% statistical power to detect this effect size between groups (a one-sided alpha = 0.05, beta = 0.20). Since recruitment was increased by 40% to compensate for loss to follow-up, poor compliance, and 1-year mortality rate, a total of 70 patients per group was required for this study.

Data are presented as number and percentage (%) for categorical variables, and mean ± standard deviation for continuous variables. Baseline patient characteristics and the results of both groups were assessed for normality using Kolmogorov-Smirnov test. Pearson’s chi-square or Fisher’s exact test were used to compare categorical variables. For continuous variables, Student’s t-test was used to compare parametric data, and Mann-Whitney U test was used to compare nonparametric data. Comparison of the percentage of patients with increased, decreased, or stable BMDs at the lumbar spine, total hip, and femoral neck was performed using chi-square test. One-way repeated measures ANOVA was used to assess the effect of time on the change in each bone turnover marker in each patient group. Statistical analysis was performed per protocol. All analyses were performed using SPSS Statistics version 18.0 (SPSS, Inc., Chicago, IL, USA). Since this study was a non-inferiority trial, a one-sided *p*-value <0.05 was regarded as statistically significant.

## Results

**Fig 1:**
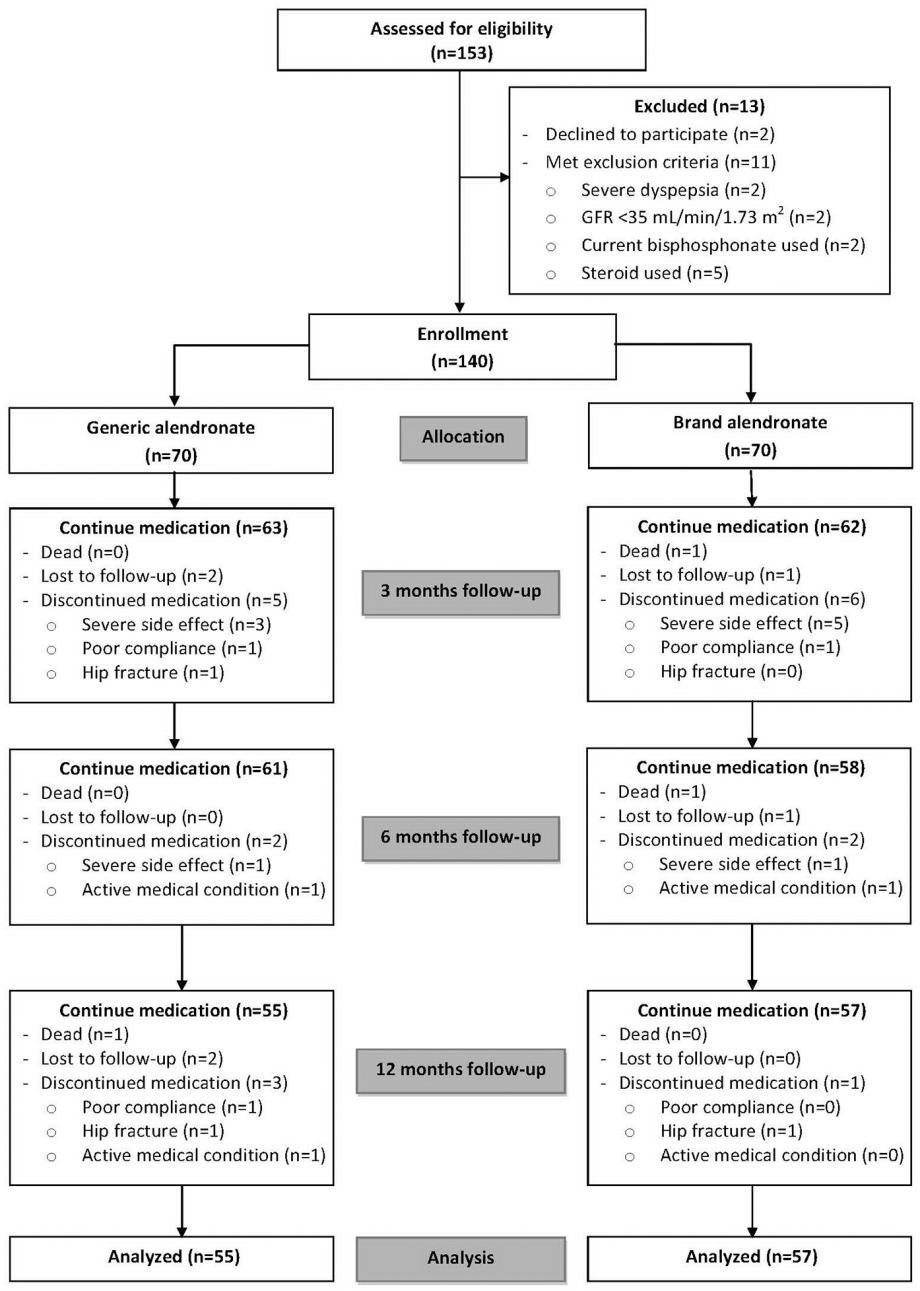
Consolidated Standards of Reporting Trials (CONSORT) diagram illustrating the flow of patients in this study.

A total of 153 patients were screened during the study period. Of 153 patients, 13 patients were excluded from the study, as follows: 2 patients for declining to participate, 2 patients with severe dyspepsia, 2 patients with estimated glomerular filtration rate <35 mL/min/1.73 m^2^, 2 patients currently using bisphosphonate, and 5 patients had history of taking glucocorticoids during the past 6 months. The remaining 140 patients were enrolled. Included participants were randomized and allocated according to the study protocol (70 patients to the generic alendronate group and 70 patients to the brand alendronate group). Three patients died during study treatment as a result of underlying medical problems. Six patients were lost to follow-up during the study period. Nineteen patients discontinued medication after the randomization process for a variety of reasons that included fracture, severe side effects, and active medical condition. One hundred and twelve patients (80%) completed the study with data available for analysis at the end of the 12-month follow-up (Fig 1).

Demographic and baseline characteristics of patients in both study groups are shown in Table 1. The mean ± standard deviation age of our study population was 73.7 ± 8.5 years. Most subjects (90.7%) were women. The predominant indication for using bisphosphonate was history of low-energy spine or hip fracture (59.3%). When comparing demographic data between the generic and brand treatment groups, there were no differences in any of the demographic or clinical characteristics between groups. Baseline BMD and laboratory investigations are presented in Table 1. At baseline, only BMD at the femoral neck and total hip was significantly different between the 2 treatment groups (*p* = 0.001 and *p* = 0.027, respectively).

**Table 1:** Patient demographic and baseline clinical and laboratory characteristics.

Clinical variables	Generic alendronate (Bonmax^®^) (n = 70)	Brand alendronate (Fosamax^®^) (n = 70)	*p*-value
Age (years)	73.7 ± 7.2	73.7 ± 9.7	0.969
Gender (Female), n (%)	61 (87.1%)	66 (94.3%)	0.145
Body mass index (kg/m^2^)	24.2 ± 4.4	23.3 ± 4.0	0.244
Charlson comorbidity index, n (%)			0.591
• 0–1	60 (85.7%)	85.7%)	
• 2–3	9 (12.9%)	14.3%)	
• >3	1 (1.4%)	0 (0.0%)	
History of fracture, n (%)			0.366
• None	19 (27.1%)	25.7%)	
• Spine	32 (45.7%)	45.7%)	
• Hip	9 (12.9%)	(11.4%)	
• Spine and hip	0 (0.0%)	4 (5.7%)	
• Others	10 (14.3%)	8 (11.4%)	
History of steroid use, n (%)	4 (5.7%)	2 (2.9%)	0.404
Indication for using bisphosphonate, n (%)			0.291
• History of low-energy spine or hip fracture	40 (57.1%)	61.4%)	
• Diagnosed as osteoporosis (T-score ≥-2.5)	22 (31.4%)	34.3%)	
• Diagnosed as osteopenia (T-score between -1.0 and -2.5) with high risk of fracture^a^	8 (11.4%)	3 (4.3%)	
EuroQoL visual analogue scale	65.3 ± 15.6	67.4 ± 15.7	0.545
Baseline bone mineral density (g/cm^2^)			
• Lumbar spine	0.852 ± 0.151	0.827 ± 0.158	0.342
• Femoral neck	0.675 ± 0.109	0.612 ± 0.101	0.001
• Total hip	0.704 ± 0.127	0.643 ± 0.122	0.027
Estimated glomerular filtration rate (mL/min/1.73 m^2^)	70.8 ± 19.5	71.3 ± 18.9	0.860
Serum total calcium level (mg/dL)	9.2 ± 0.3	9.4 ± 0.5	0.117
Serum albumin level (g/dL)	4.1 ± 0.3	4.1 ± 0.3	0.742
Serum phosphate level (mg/dL)	3.5 ± 0.4	3.6 ± 0.5	0.110
Serum parathyroid hormone level (pg/mL)	52.9 ± 20.4	48.8 ± 18.0	0.221
Serum 25(OH)D level (ng/mL)	31.7 ± 9.2	33.5 ± 9.2	0.252
Serum alkaline phosphatase level (U/L)	79.3 ± 27.6	85.0 ± 28.6	0.136
Serum C-telopeptide (ng/mL)	0.6 ± 0.2	0.7 ± 0.3	0.125
Serum total procollagen type I amino-terminal propeptide (P1NP) (ng/mL)	64.4 ± 26.3	77.0 ± 47.8	0.232

**Abbreviation**: 25(OH)D, 25-hydroxyvitamin D. Data are presented as mean ± standard deviation unless otherwise specified. *p*-value <0.05 indicates statistical significance

^a^ Fracture risk was determined from a 10-year hip fracture probability ≥3% or a 10-year major osteoporosis-related fracture probability ≥20% based on FRAX^®^

**Fig 2:**
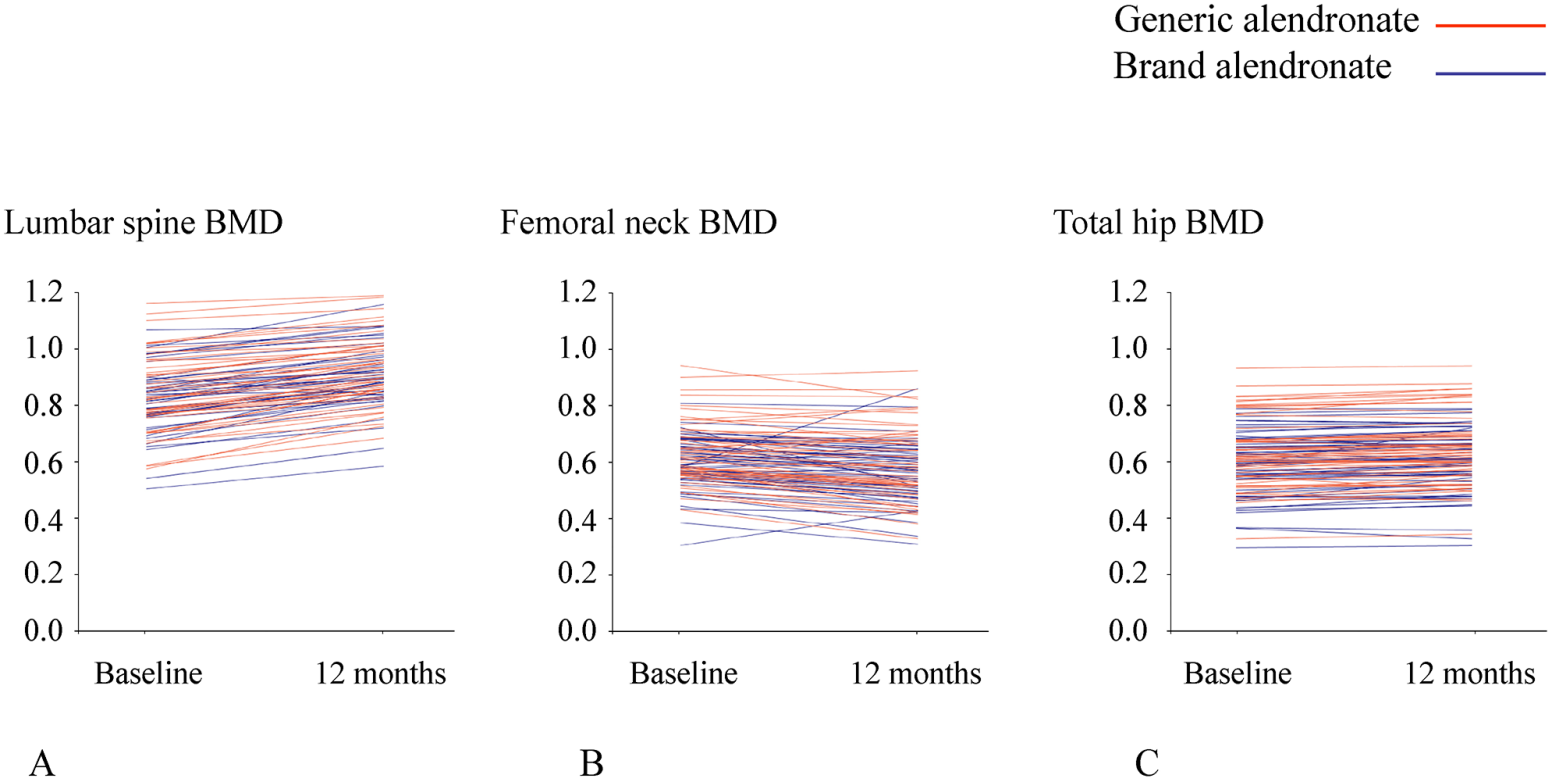
“Spaghetti plot” graphs showing changes in BMD for each patient at the (A) lumbar spine, (B) femoral neck, and (C) total hip.

The mean BMD increased from baseline to 1-year post-treatment at all sites in both groups (Table 2). Spaghetti plot graphs showing change in BMD at all 3 sites in each patient are given in Fig 2. At 1-year post-treatment, lumbar spine BMD increased an average of 5.4% and 5.5% compared with baseline in the generic and brand alendronate groups, respectively. There was no difference in percentage change in lumbar spine BMD between groups (*p* = 0.900). At 1 year, mean percentage change in total hip BMD increased approximately 2.5% in both treatment groups. Similar to lumbar spine BMD, there was no difference in percentage change in total hip BMD between groups (*p* = 0.952). Although femoral neck BMD at 1-year post-treatment increased by an average of 1.9% and 4.4% relative to baseline in the generic and brand alendronate groups, respectively, the difference was not statistically significant (*p* = 0.163) (Table 2).

**Table 2:** Changes in bone mineral density after one year of treatment with generic or brand alendronate.

Bone mineral density measurement (g/cm^2^)	Generic alendronate (Bonmax^®^)				Brand alendronate (Fosamax^®^)				Between groups *p*-value
	Pairs^a^	Mean ± standard deviation	95% CI	Within group *p*-value	Pairs^a^	Mean ± standard deviation	95% CI	Within group *p*-value	
Lumbar spine									
• At baseline	46	0.840 ± 0.140	0.798–0.882		44	0.818 ± 0.121	0.781–0.855		0.425
• At 1 year	46	0.883 ± 0.138	0.842–0.924	<0.001	44	0.863 ± 0.131	0.823–0.903	<0.001	0.469
• Percentage change at 1 year	46	5.39 ± 4.83	3.96–6.82		44	5.54 ± 6.39	3.60–7.48		0.900
Femoral neck									
• At baseline	52	0.671 ± 0.112	0.640–0.702		56	0.630 ± 0.093	0.605–0.655		0.039
• At 1 year	52	0.683 ± 0.116	0.651–0.715	0.001	56	0.653 ± 0.089	0.629–0.677	0.001	0.128
• Percentage change at 1 year	52	1.85 ± 6.16	0.14–3.57		56	4.43 ± 11.87	1.25–7.61		0.163
Total hip									
• At baseline	54	0.710 ± 0.125	0.676–0.744		57	0.664 ± 0.111	0.635–0.693		0.044
• At 1 year	54	0.727 ± 0.126	0.693–0.761	<0.001	57	0.679 ± 0.112	0.649–0.709	<0.001	0.039
• Percentage change at 1 year	54	2.52 ± 3.51	1.56–3.48		57	2.48 ± 4.56	1.27–3.69		0.952

**Abbreviation**: 95% CI, 95% confidence interval. *p*-value <0.05 indicates statistical significance

^a^ Pairs = patients with data available at baseline and at the 1-year follow-up

When classifying the magnitude of BMD change at each site into three types based on the least significant change (increased, stable, or decreased BMD), we found that >85% of patients in both groups had stable or increased BMD after 1-year of alendronate treatment (Table 3). There were no differences in the percentage of patients who were classified as increased, stable, or decreased BMD after 1 year of treatment between generic and brand alendronate patients (*p*-value range: 0.475–0.859). Similar to BMD, there was no difference in EQ-VAS at 1-year post-treatment between the 2 groups. The mean EQ-VAS at 1-year post-treatment was 79.4 ± 13.6 and 80.3 ± 14.2 for the generic and brand alendronate groups, respectively (*p* = 0.588).

**Table 3:** Number and percentage of patients with stable, increased, or decreased bone mineral densities after one year of treatment with generic or brand alendronate.

Bone mineral density	Generic alendronate (Bonmax^®^)			Brand alendronate (Fosamax^®^)			Between groups *p*-value
	Decreased	Stable	Increased	Decreased	Stable	Increased	
Lumbar spine^a^	1 (2.2%)	13 (28.3%)	32 (69.6%)	2 (4.5%)	14 (31.8%)	28 (63.6%)	0.743
Femoral neck^b^	6 (11.5%)	32 (61.5%)	14 (26.9%)	3 (5.4%)	35 (62.5%)	18 (32.1%)	0.475
Total hip^b^	1 (1.9%)	41 (75.9%)	12 (22.2%)	2 (3.5%)	42 (73.7%)	13 (22.8%)	0.859

Data are presented as number and percentage; *p*-value <0.05 indicates statistical significance

^a^ Lumbar spine BMD was categorized as decreased if the amount of BMD loss was ≥3%, stable if the percentage change was between -3 and +3%, and increased if the amount of BMD gain was ≥3%

^b^ Femoral neck and total hip BMD values were categorized as decreased if the amount of BMD loss was ≥5%, stable if the percentage change was between -5 and +5%, and increased if the amount of BMD gain was ≥5%

**Fig 3:**
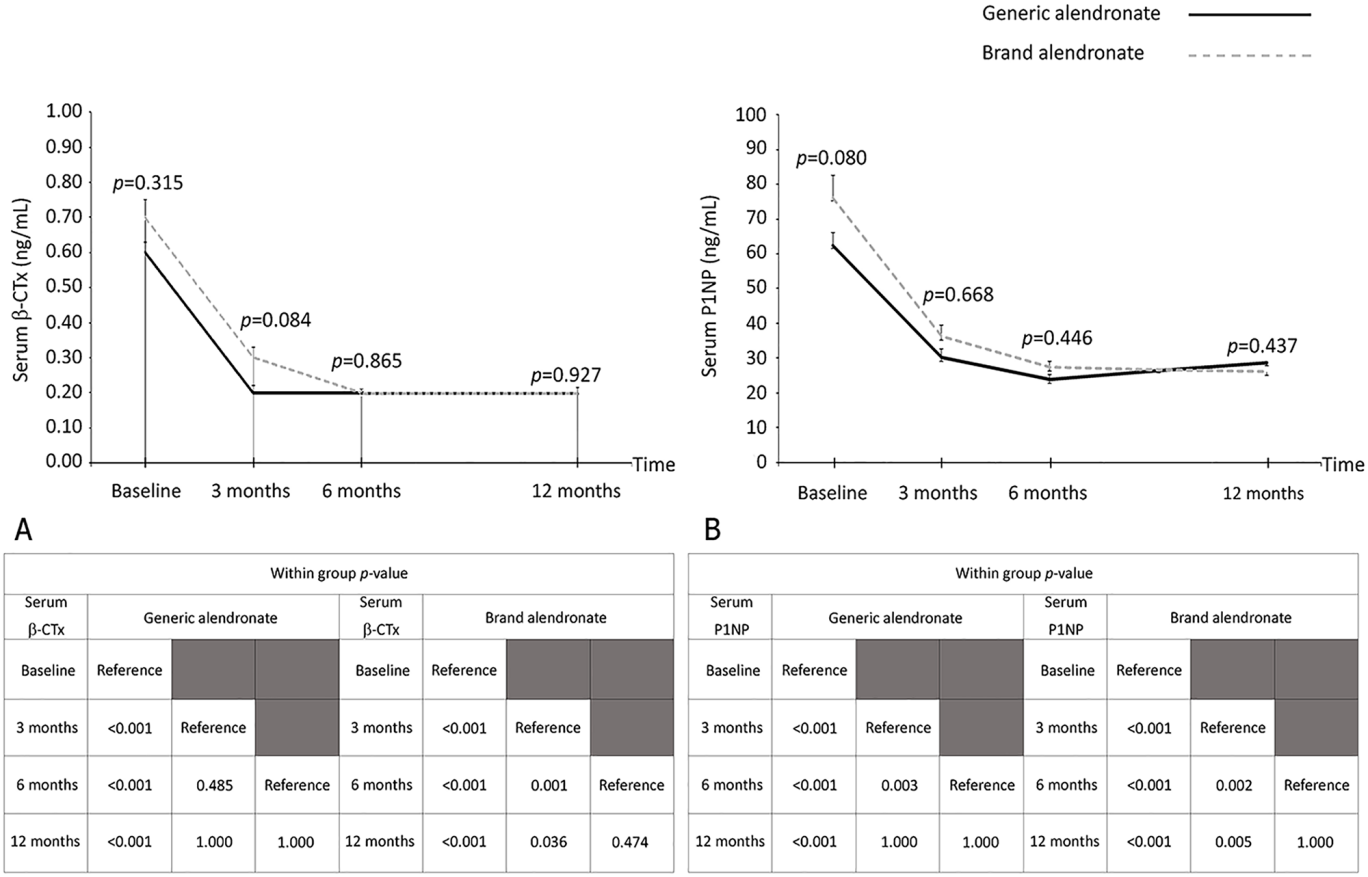
Mean values of biochemical bone markers, and within group and between group *p*-values. (A) serum β-isomerized C-terminal telopeptide (β-CTx); and, (B) serum total procollagen type 1 amino-terminal propeptide (P1NP) for the generic and brand alendronate patient groups before treatment (baseline) and at 3, 6, and 12 months after treatment. The error bars indicate standard error. The *p*-values in the graphs compare levels between the generic and brand alendronate patient groups, while within group *p*-values are shown in the corresponding table below each graph (*p*-value <0.05 indicates statistical significance).

The mean value of the biochemical bone marker for bone resorption (β-CTx) in both the generic and brand alendronate groups decreased from baseline at all follow-up time points, with the lowest level at 6 months after treatment (*p* < 0.001). Similar to β-CTx, the mean serum P1NP level in the generic group decreased from baseline at all follow-up time points, with the lowest levels at 6 months after treatment (*p* < 0.001). Conversely, in the brand alendronate group, mean serum P1NP level decreased from baseline at all time points, with the lowest level at 12 months after treatment (*p* < 0.001). There were no differences in serum β-CTx and P1NP levels between the generic and brand alendronate groups at all time points (*p* > 0.08) (Fig 3).

Table 4 shows the proportion of adverse events in each treatment group. The most common adverse events found after taking either formulations of alendronate were myalgia and arthralgia. The rate of adverse events was similar between the generic and brand alendronate groups (*p* = 0.471). Five patients had new fractures during the study period. Of those, there were 2 hip fractures in the generic alendronate group and 1 hip fracture in the brand alendronate group. A new clinical vertebral fracture and a distal end radius fracture also occurred in the brand alendronate group. Adverse events were severe enough to warrant discontinuation of alendronate in 6 patients (8.5%) and 4 patients (5.7%) in the generic and brand alendronate groups, respectively (*p* = 0.512).

**Table 4:** Type and frequency of observed adverse events.

Adverse events	Generic alendronate (Bonmax^®^)		Brand alendronate (Fosamax^®^)		*p*-value
	(n = 70)	95% CI	(n = 70)	95% CI	
Total number of side effects	21 (30%)	0.205–0.415	25 (35.7%)	0.255–0.474	0.471
• Myalgia and arthralgia	10 (14.3%)	0.079–0.243	16 (22.9%)	0.146–0.340	
• Gastrointestinal^a^	5 (7.1%)	0.031–0.157	7 (10%)	0.049–0.192	
• Fever	2 (2.9%)	0.008–0.098	-	0.000–0.052	
• Urticarial rash	1 (1.4%)	0.003–0.077	-	0.000–0.052	
• Hypocalcemia	2 (2.9%)	0.008–0.098	1 (1.4%)	0.003–0.077	
• Dizziness	1 (1.4%)	0.003–0.077	1 (1.4%)	0.003–0.077	
Total number of new fractures	2 (2.9%)	0.008–0.098	3 (4.3%)	0.015–0.119	1.000
• Hip fracture	2 (2.9%)	0.008–0.098	1 (1.4%)	0.003–0.077	
• Vertebral fracture	-	0.000–0.052	1 (1.4%)	0.003–0.077	
• Distal end radius fracture	-	0.000–0.052	1 (1.4%)	0.003–0.077	
Patients who discontinued medication due to side effect	6 (8.6%)	0.040–0.175	4 (5.7%)	0.022–0.138	0.512

**Abbreviation**: 95% CI, 95% confidence interval. Data are presented as number and percentage; *p*-value <0.05 indicates statistical significance.

^a^ Gastrointestinal side effects were dyspepsia, gastroesophageal reflux disease, and abdominal cramping

## Discussion

In general, generic drugs contain active pharmaceutical ingredients similar to those used in original brand name drugs; therefore, preclinical studies and clinical trials on animals and patients to prove the safety and efficacy of generic drug products are not required [11,22]. Alternatively, bioequivalence studies are required that demonstrate safety and efficacy comparability between the generic and its brand predecessor [11]. Although therapeutically equivalent products are expected to have the same safety and efficacy profiles, many generic drugs are not administered under the same conditions [12]. In Thailand, many generic forms of alendronate are available. Various preparations of alendronate 70 mg per week contain different ratios of alendronate monosodium and alendronate sodium trihydrate. Changes in these content ratios may cause differences in pharmacokinetics and pharmacodynamics, which may result in different outcomes regarding efficacy and side effects. Most of these generic drugs provide comparative bioequivalence data with the original drug, but none has been compared in terms of therapeutic equivalence or safety/tolerability to brand alendronate.

In this study, both generic and brand alendronate showed significant increase in BMD at the lumbar spine, total hip, and femoral neck at 1-year post-treatment. Mean BMD increased 5.5% and 2.5% in both groups at the lumbar spine and total hip, respectively. BMD response at all sites was consistent with results of previous studies, with lumbar spine as the site with the largest change among all skeletal sites [19,20]. The percentage of patients with change in total hip or femoral neck BMD ≥5% or lumbar spine BMD ≥3% was evaluated as another predictor of relative antiresorptive potency. These 3% and 5% changes were chosen because they correspond with the least significant change necessary to be 95% confident that the improvement in BMD in an individual patient is genuine [23]. Changes equal to or greater than this magnitude have been shown to predict substantial anti-fracture benefit with alendronate. It is important, however, to point out that even patients with an apparent loss of BMD during bisphosphonate therapy have been shown to have some fracture risk reduction [24]. Our study showed that there was no difference in the percentage of patients who were classified as increased, stable, or decreased BMD after 1 year of treatment between generic and brand alendronate. Thus, both forms of alendronate provided similar clinical efficacy regarding BMD responses.

The rapid decrease in both bone turnover markers (c-CTx and P1NP) during the first 3 months was achieved with both alendronate formulations, followed by a more gradual decrease at 6 and 12 months, which is comparable to previous findings in postmenopausal women with osteoporosis who received treatment with alendronate [25,26]. The lowest level of β-CTx was at 6 months after treatment in both treatment groups, while the lowest level of serum P1NP was at 6 and 12 months in the generic and brand alendronate groups, respectively. These differences in time points at which serum P1NP was at its lowest level may be related to differential pharmacokinetic profiles between generic and brand alendronate [27,28]. Although time achieved the lowest serum P1NP level was faster in the generic alendronate group, the magnitude of reduction was similar between generic and brand alendronate. We, therefore, consider the difference in time of the lowest level of bone formation marker between the 2 treatment groups to be clinically non-relevant.

Alendronate is slowly absorbed and carries a high risk of esophageal irritation [29,30]. Previous studies reported that generic alendronate might not be as well-tolerated as brand alendronate. Grima DT, *et al*. [10] reported that after introduction of an automatic substitution to generic alendronate in Canada, patients who were previously stable on brand alendronate experienced an increase in adverse events, particularly gastrointestinal side effects. Another group [28] conducted a randomized double-blind cross-over study in postmenopausal women and found that generic alendronate (Accord^®^) caused significantly higher abdominal pain scores in the first 4 weeks of treatment, although there was no significant difference in the overall tolerance between the generic and brand alendronate groups. In contrast to the results of previous studies, there were no significant differences in total adverse events in our study. The most frequently observed side effects in this study were myalgia and arthralgia, instead of the gastrointestinal-related problems described in the following study. Similar to our results, Orwoll ES, *et al*. [31] reported that the most frequently occurring adverse events after bisphosphonate administration (both intravenous and oral formulations) were pyrexia, myalgia, arthralgia, influenza-like illness, malaise, and backache. These symptoms were transient, however, and generally did not lead to discontinuation of bisphosphonate administration.

Drug compliance is another concern in patients who take generic alendronate due to the higher rate of side effects that can motivate patients to stop taking the medication [32]. Our controlled-trial study did not observe a negative effect on either compliance or adherence between the two treatment groups. Similar to our findings, a study [11] reported that adherence was similar between patients receiving generic (apo-alendronate^®^) and brand alendronate.

This study has several limitations. First, the patients were not blinded to their assigned treatment medication. Given that generic and brand alendronate are different regarding size and shape of the pill and the package, it is not feasible to blind patients to their assigned treatment. Although this is a potential source of bias, we used BMD and bone turnover markers as outcomes to determine treatment efficacy. Blinding or not blinding patients should not affect changes in BMD and bone turnover markers in patients of either study group. Second, we investigated only one type of generic alendronate (Bonmax^®^), which is the generic available at our center. As such, the results of our study are not generalizable to other generic alendronate drugs. Third, our sample size was calculated based on changes in BMD after 1 year of treatment. Thus, our sample size was too small to detect other clinically important secondary outcome measures. Furthermore, our sample size was too small to detect difference in fracture incidence between the treatment groups. Ideally, new fractures should be used as outcomes to determine efficacy of an antiosteoporosis agent. However, since the incidence of new fractures is much reduced after bisphosphonate treatment, this will increase the number of sample sizes required for such a study. Nevertheless, our study used validated, surrogated endpoints (BMD and bone markers), which are the best alternative when a clinically relevant outcome (i.e., fracture) is not available [33,34]. Finally, the duration of follow-up was short. A similar study design with longer BMD follow-up and that includes fracture data is warranted.

In conclusion, both generic and brand alendronate increased BMD at all sites and reduced biochemical bone markers. These two alendronate formulations yielded similar clinical efficacy. Both forms of alendronate were well-tolerated, with no significant difference in the number of patients with adverse events or discontinuation due to treatment side effects. Based on these findings, Bonmax^®^ (the generic brand used in this study) is a viable alternative to the original brand of alendronate, which is an anti-osteoporosis agent with long-term efficacy and safety.

## Supporting information

S1 File Study protocol in Thai language. (PDF)

S2 File Study protocol in English language. (PDF)

S1 Checklist CONSORT checklist. (PDF)

## References

[1] U.S. Department of Health and Human Services. Bone Health and Osteoporosis: A Report of the Surgeon General. Rockville, MD: U.S. Department of Health and Human Services, Office of the Surgeon General; 2004.

[2] Eisman JA, Bogoch ER, Dell R, Harrington JT, McKinney RE Jr., McLellan A, et al Making the first fracture the last fracture: ASBMR task force report on secondary fracture prevention. J Bone Miner Res. 2012;27(10):2039–46. doi: 10.1002/jbmr.1698 22836222 10.1002/jbmr.1698

[3] Papaioannou A, Kennedy CC, Ioannidis G, Sawka A, Hopman WM, Pickard L, et al The impact of incident fractures on health-related quality of life: 5 years of data from the Canadian Multicentre Osteoporosis Study. Osteoporos Int. 2009;20(5):703–14. doi: 10.1007/s00198-008-0743-7 18802659 10.1007/s00198-008-0743-7 PMC5101052

[4] Tarride JE, Hopkins RB, Leslie WD, Morin S, Adachi JD, Papaioannou A, et al The burden of illness of osteoporosis in Canada. Osteoporos Int. 2012;23(11):2591–600. doi: 10.1007/s00198-012-1931-z 22398854 10.1007/s00198-012-1931-z PMC3483095

[5] Pongchaiyakul C, Leerapun T, Wongsiri S, Songpattanasilp T, Taechakraichana N. Value and validation of RCOST and TOPF clinical practice guideline for osteoporosis treatment. J Med Assoc Thai. 2012;95(12):1528–35. 23390783

[6] Sanderson J, Martyn-St James M, Stevens J, Goka E, Wong R, Campbell F, et al Clinical effectiveness of bisphosphonates for the prevention of fragility fractures: A systematic review and network meta-analysis. Bone. 2016;89:52–8. doi: 10.1016/j.bone.2016.05.013 27262775 10.1016/j.bone.2016.05.013

[7] Black DM, Cummings SR, Karpf DB, Cauley JA, Thompson DE, Nevitt MC, et al Randomised trial of effect of alendronate on risk of fracture in women with existing vertebral fractures. Fracture Intervention Trial Research Group. Lancet. 1996;348(9041):1535–41. 8950879 10.1016/s0140-6736(96)07088-2

[8] Bone HG, Hosking D, Devogelaer JP, Tucci JR, Emkey RD, Tonino RP, et al Ten years' experience with alendronate for osteoporosis in postmenopausal women. N Engl J Med. 2004;350(12):1189–99. doi: 10.1056/NEJMoa030897 15028823 10.1056/NEJMoa030897

[9] Hassler N, Gamsjaeger S, Hofstetter B, Brozek W, Klaushofer K, Paschalis EP. Effects of long-term alendronate treatment on postmenopausal osteoporosis bone material properties. Osteoporos Int. 2015;26(1):339–52. doi: 10.1007/s00198-014-2929-5 25315260 10.1007/s00198-014-2929-5

[10] Grima DT, Papaioannou A, Airia P, Ioannidis G, Adachi JD. Adverse events, bone mineral density and discontinuation associated with generic alendronate among postmenopausal women previously tolerant of brand alendronate: a retrospective cohort study. BMC Musculoskelet Disord. 2010;11:68 doi: 10.1186/1471-2474-11-68 20388226 10.1186/1471-2474-11-68 PMC2867835

[11] Lai PS, Chua SS, Chong YH, Chan SP. The effect of mandatory generic substitution on the safety of alendronate and patients' adherence. Curr Med Res Opin. 2012;28(8):1347–55. doi: 10.1185/03007995.2012.708326 22746354 10.1185/03007995.2012.708326

[12] Brown JP, Davison KS, Olszynski WP, Beattie KA, Adachi JD. A critical review of brand and generic alendronate for the treatment of osteoporosis. Springerplus. 2013;2:550 doi: 10.1186/2193-1801-2-550 25674402 10.1186/2193-1801-2-550 PMC4320211

[13] Kanis JA, Oden A, Johnell O, Johansson H, De Laet C, Brown J, et al The use of clinical risk factors enhances the performance of BMD in the prediction of hip and osteoporotic fractures in men and women. Osteoporos Int. 2007;18(8):1033–46. doi: 10.1007/s00198-007-0343-y 17323110 10.1007/s00198-007-0343-y

[14] Kanis JA, Johnell O, Oden A, Johansson H, McCloskey E. FRAX and the assessment of fracture probability in men and women from the UK. Osteoporos Int. 2008;19(4):385–97. doi: 10.1007/s00198-007-0543-5 18292978 10.1007/s00198-007-0543-5 PMC2267485

[15] Savaridas T, Wallace RJ, Salter DM, Simpson AHRW. Do bisphosphonates inhibit direct fracture healing? A laboratory investigation using an animal model. Bone Joint J. 2013;95-B(9):1263–8. doi: 10.1302/0301-620X.95B9.31562 23997143 10.1302/0301-620X.95B9.31562

[16] Baim S, Wilson CR, Lewiecki EM, Luckey MM, Downs RW Jr., Lentle BC. Precision assessment and radiation safety for dual-energy X-ray absorptiometry: position paper of the International Society for Clinical Densitometry. J Clin Densitom. 2005;8(4):371–8. 16311420 10.1385/jcd:8:4:371

[17] Tidermark J, Zethraeus N, Svensson O, Tornkvist H, Ponzer S. Femoral neck fractures in the elderly: functional outcome and quality of life according to EuroQol. Qual Life Res. 2002;11(5):473–81. 12113394 10.1023/a:1015632114068

[18] Tidermark J, Bergstrom G, Svensson O, Tornkvist H, Ponzer S. Responsiveness of the EuroQol (EQ 5-D) and the SF-36 in elderly patients with displaced femoral neck fractures. Qual Life Res. 2003;12(8):1069–79. 14651424 10.1023/a:1026193812514

[19] Rosen CJ, Hochberg MC, Bonnick SL, McClung M, Miller P, Broy S, et al Treatment with once-weekly alendronate 70 mg compared with once-weekly risedronate 35 mg in women with postmenopausal osteoporosis: a randomized double-blind study. J Bone Miner Res. 2005;20(1):141–51. doi: 10.1359/JBMR.040920 15619680 10.1359/JBMR.040920

[20] Paggiosi MA, Peel N, McCloskey E, Walsh JS, Eastell R. Comparison of the effects of three oral bisphosphonate therapies on the peripheral skeleton in postmenopausal osteoporosis: the TRIO study. Osteoporos Int. 2014;25(12):2729–41. doi: 10.1007/s00198-014-2817-z 25074351 10.1007/s00198-014-2817-z

[21] Zülfįkaroğlu E, Kiliç S, Eserdağ S, Batioğlu S. Effects of Alendronate and Raloxifene on Bone Density and Bone Turnover Markers in Postmenopausal Women. Gynecol Obstet Reprod Med. 2011;17:34–8.

[22] Lietzan EK. A brief history of 180-day exclusivity under the Hatch-Waxman Amendments to the Federal Food, Drug, and Cosmetic Act. Food Drug Law J. 2004;59(2):287–323. 15298013

[23] Bonnick SL, Johnston CC Jr., Kleerekoper M, Lindsay R, Miller P, Sherwood L, et al Importance of precision in bone density measurements. J Clin Densitom. 2001;4(2):105–10. 11477303 10.1385/jcd:4:2:105

[24] Cummings SR, Karpf DB, Harris F, Genant HK, Ensrud K, LaCroix AZ, et al Improvement in spine bone density and reduction in risk of vertebral fractures during treatment with antiresorptive drugs. Am J Med. 2002;112(4):281–9. 11893367 10.1016/s0002-9343(01)01124-x

[25] Greenspan SL, Rosen HN, Parker RA. Early changes in serum N-telopeptide and C-telopeptide cross-linked collagen type 1 predict long-term response to alendronate therapy in elderly women. J Clin Endocrinol Metab. 2000;85(10):3537–40. doi: 10.1210/jcem.85.10.6911 11061497 10.1210/jcem.85.10.6911

[26] Vasikaran S, Eastell R, Bruyere O, Foldes AJ, Garnero P, Griesmacher A, et al Markers of bone turnover for the prediction of fracture risk and monitoring of osteoporosis treatment: a need for international reference standards. Osteoporos Int. 2011;22(2):391–420. doi: 10.1007/s00198-010-1501-1 21184054 10.1007/s00198-010-1501-1

[27] Dansereau RJ, Crail DJ, Perkins AC. In vitro disintegration studies of weekly generic alendronate sodium tablets (70 mg) available in the US. Curr Med Res Opin. 2009;25(2):449–52. doi: 10.1185/03007990802648903 19192989 10.1185/03007990802648903

[28] van den Bergh JP, Bouts ME, van der Veer E, van der Velde RY, Janssen MJ, Geusens PP, et al Comparing tolerability and efficacy of generic versus brand alendronate: A randomized clinical study in postmenopausal women with a recent fracture. PLoS One. 2013;8(10):e78153 doi: 10.1371/journal.pone.0078153 24205135 10.1371/journal.pone.0078153 PMC3804551

[29] Watts N, Freedholm D, Daifotis A. The clinical tolerability profile of alendronate. Int J Clin Pract Suppl. 1999;101:51–61. 12669741

[30] Biswas PN, Wilton LV, Shakir SA. Pharmacovigilance study of alendronate in England. Osteoporos Int. 2003;14(6):507–14. doi: 10.1007/s00198-003-1399-y 12730757 10.1007/s00198-003-1399-y

[31] Orwoll ES, Miller PD, Adachi JD, Brown J, Adler RA, Kendler D, et al Efficacy and safety of a once-yearly i.v. Infusion of zoledronic acid 5 mg versus a once-weekly 70-mg oral alendronate in the treatment of male osteoporosis: a randomized, multicenter, double-blind, active-controlled study. J Bone Miner Res. 2010;25(10):2239–50. doi: 10.1002/jbmr.119 20499357 10.1002/jbmr.119

[32] Strom O, Landfeldt E. The association between automatic generic substitution and treatment persistence with oral bisphosphonates. Osteoporos Int. 2012;23(8):2201–9. doi: 10.1007/s00198-011-1850-4 22120909 10.1007/s00198-011-1850-4

[33] Bucher HC, Guyatt GH, Cook DJ, Holbrook A, McAlister FA. Users' guides to the medical literature: XIX. Applying clinical trial results. A. How to use an article measuring the effect of an intervention on surrogate end points. Evidence-Based Medicine Working Group. JAMA. 1999;282(8):771–8. 10463714 10.1001/jama.282.8.771

[34] McAlister FA, Laupacis A, Wells GA, Sackett DL. Users' Guides to the Medical Literature: XIX. Applying clinical trial results B. Guidelines for determining whether a drug is exerting (more than) a class effect. JAMA. 1999;282(14):1371–7. 10527185 10.1001/jama.282.14.1371

