# Supplementary material for: Randomized clinical trial comparing efficacy and safety of brand versus generic alendronate (Bonmax®) for osteoporosis treatment
Source: PLoS One. 2017 Jul 5;12(7):e0180325. doi: 10.1371/journal.pone.0180325 (PMC5498028; doi:10.1371/journal.pone.0180325)
Supplement: S1 File — (PDF) [file pone.0180325.s001.pdf]

แบบโครงร่างงานวิจัย

การศึกษาเปรียบเทียบแบบสุ่มของประสิทธิภาพและความปลอดภัยของการใช้ยาอะเลนโดรเนตต้นแบบและสามัญในการรักษาโรคกระดูกพรุน

รศ.นพ.อาศิส อุนนะนันทน์

นพ.อรรถกร จารุศรีวรรณา

ศ.นพ.ภานุพันธ์ ทรงเจริญ

ภาควิชาศัลยศาสตร์ออร์โธปิดิคส์และกายภาพบำบัด

คณะแพทยศาสตร์ศิริราชพยาบาล มหาวิทยาลัยมหิดล

สารบัญ

ข้อมูลทั่วไปของโครงการและผู้วิจัย (Protocol identification and Investigator) 3

1. ชื่อโครงการวิจัย 3
2. ชื่อหัวหน้าโครงการวิจัย 3
3. ผู้วิจัยร่วมทั้งหมด 3
4. แหล่งทุนสนับสนุนการวิจัย (Research funding) 3
5. สถานที่ทำวิจัย 4
6. ระยะเวลาที่ทำโครงการวิจัย 4
7. โครงการนี้เป็นส่วนหนึ่งของการศึกษาเพื่อปริญญาบัตร วุฒิบัตร หรือการศึกษาอิสระ (Independent study) 4
8. สรุปโครงร่างวิจัย 4
   - 8.1 หลักการและเหตุผลที่ต้องทำวิจัย (Background/Rationale) 4
   - 8.2 วัตถุประสงค์ของการวิจัย (Objective) 5
   - 8.3 ประเภทของโครงการวิจัย (Research type) 6
   - 8.4 การออกแบบการวิจัย (Research design) 7
   - 8.5 ผู้ร่วมวิจัย/อาสาสมัคร (Research subjects) 7
   - 8.6 กระบวนการวิจัย (Research process) 9
   - 8.7 กระบวนการเก็บข้อมูล (Data collection process) 10
   - 8.8 การวัดผล/การวิเคราะห์ผลการวิจัย (Outcome measurement/Data analysis) 10
   - 8.9 หลักฐาน ข้อมูล หรือเอกสารอ้างอิง (References) 11

ข้อพิจารณาด้านจริยธรรมการวิจัยในคน (Ethical consideration) 12

9. ลักษณะผู้ร่วมวิจัย/อาสาสมัคร 12
10. การใช้ข้อมูลและการเก็บชีววัตถุของผู้ร่วมวิจัย/อาสาสมัคร 12
11. กระบวนการเชิญชวนให้เข้าร่วมการวิจัย (Recruitment process) 12
    - 11.1 สถานที่ 12
    - 11.2 กระบวนการ 12
12. กระบวนการขอความยินยอมให้เข้าร่วมการวิจัย (Informed consent process) 12
13. ประโยชน์ที่คาดว่าจะได้รับจากการวิจัย 13
14. ผลกระทบที่อาจจะเกิดแก่ผู้ร่วมวิจัย/อาสาสมัคร 13
15. เกี่ยวข้องหรืออาจมีผลกระทบกระเทือนต่อศาสนา ความเชื่อ ขนบธรรมเนียมประเพณีหรือวัฒนธรรมอันดีงาม ชื่อเสียงของสถาบัน ท้องถิ่นหรือประเทศที่ทำการวิจัยอยู่หรือไม่ 13
16. วิธีการปกป้องความลับหรือข้อมูลส่วนตัวของผู้ร่วมวิจัย/อาสาสมัคร 13

Case record form 14

ข้อมูลทั่วไปของโครงการและผู้วิจัย (Protocol identification and Investigator)

1. **ชื่อโครงการวิจัย**

การศึกษาเปรียบเทียบแบบสุ่มของประสิทธิภาพและความปลอดภัยของการใช้ยาอะเลนโดรเนตต้นแบบและสามัญในการรักษาโรคกระดูกพรุน

2. **ชื่อหัวหน้าโครงการวิจัย** รศ.นพ.อาศิส อุนนะนันทน์

อาจารย์ ตำแหน่งวิชาการ รองศาสตราจารย์

สังกัด ภาควิชาศัลยศาสตร์ออร์โธปิดิคส์และกายภาพบำบัด คณะแพทยศาสตร์ศิริราชพยาบาล มหาวิทยาลัยมหิดล

สถานที่ทำงาน/ติดต่อ ภาควิชาศัลยศาสตร์ออร์โธปิดิคส์และกายภาพบำบัด คณะแพทยศาสตร์ศิริราชพยาบาล มหาวิทยาลัยมหิดล

หมายเลขโทรศัพท์ที่ติดต่อได้ทั้งในและนอกเวลาราชการ 0-2419-7968-9

E-mail address: uaasis@gmail.com, siaun@mahidol.ac.th

3. **ผู้วิจัยร่วมทั้งหมด**

ชื่อผู้วิจัยร่วม นพ.อรรถกร จารุศรีวรรณา

แพทย์ประจำบ้าน

สังกัด ภาควิชาศัลยศาสตร์ออร์โธปิดิคส์และกายภาพบำบัด คณะแพทยศาสตร์ศิริราชพยาบาล มหาวิทยาลัยมหิดล

สถานที่ทำงาน/ติดต่อ ภาควิชาศัลยศาสตร์ออร์โธปิดิคส์และกายภาพบำบัด คณะแพทยศาสตร์ศิริราชพยาบาล มหาวิทยาลัยมหิดล

หมายเลขโทรศัพท์ที่ติดต่อได้ทั้งในและนอกเวลาราชการ 0-2419-7968-9

E-mail address: wonton2ton@hotmail.com

ชื่อผู้วิจัยร่วม ศ.นพ.ภานุพันธ์ ทรงเจริญ

อาจารย์ ตำแหน่งวิชาการ ศาสตราจารย์

สังกัด ภาควิชาศัลยศาสตร์ออร์โธปิดิคส์และกายภาพบำบัด คณะแพทยศาสตร์ศิริราชพยาบาล มหาวิทยาลัยมหิดล

สถานที่ทำงาน/ติดต่อ ภาควิชาศัลยศาสตร์ออร์โธปิดิคส์และกายภาพบำบัด คณะแพทยศาสตร์ศิริราชพยาบาล มหาวิทยาลัยมหิดล

หมายเลขโทรศัพท์ที่ติดต่อได้ทั้งในและนอกเวลาราชการ 0-2419-7968-9

E-mail address: panupan.son@mahidol.ac.th

4. **แหล่งทุนสนับสนุนการวิจัย** (Research funding)

❑ ไม่มีทุน

☑ มีทุน ระบุ ทุนแพทยสมาคมแห่งประเทศไทย (กองทุนวิจัย “นายแพทย์ปราเสริฐ ปราสาททองโอสถ”)

5. **สถานที่ทำวิจัย**

☑ Single center ระบุ โรงพยาบาลศิริราช คณะแพทยศาสตร์ศิริราชพยาบาล มหาวิทยาลัยมหิดล กรุงเทพฯ

❑ Multiple centers ระบุ

6. **ระยะเวลาที่ทำโครงการวิจัย**

ตลอดโครงการ 3 ปี 2 เดือน

ระยะเวลาเก็บข้อมูล 2 ปี 6 เดือน (ระยะเวลาที่ใช้ในการรวบรวมผู้เข้าร่วมวิจัยได้ทั้งหมด)

**กิจกรรม** (Activity)	**ช่วงเวลา**
การเตรียมการ การขอทุน และขอการรับรองโครงการจากคณะกรรมการจริยธรรมการวิจัยในคน	1 กุมภาพันธ์ 2557 ถึง 30 เมษายน 2557
การเก็บข้อมูล	1 พฤษภาคม 2557 ถึง 31 ตุลาคม 2559
การวิเคราะห์ข้อมูล	1 พฤศจิกายน 2559 ถึง 31 ธันวาคม 2559
Manuscript preparation and submission	1 มกราคม 2560 ถึง 31 มีนาคม 2560

7. **โครงการนี้เป็นส่วนหนึ่งของการศึกษาเพื่อปริญญาบัตร วุฒิบัตร หรือการศึกษาอิสระ** (Independent study)

☑ ไม่ใช่

❑ ใช่ ระบุ

8. **สรุปโครงร่างวิจัย**

8.1 หลักการและเหตุผลที่ต้องทำวิจัย (Background/Rationale)

โรคกระดูกพรุนเป็นโรคที่เกิดจากการที่ร่างกายมีมวลกระดูกลดลง ทำให้โครงสร้างของกระดูกบางลง จึงส่งผลให้กระดูกมีความเปราะและแตกหักได้ง่าย ผู้ป่วยจึงมีคุณภาพชีวิตที่ลดลง และอาจเกิดภาวะแทรกซ้อนต่างๆ จนนำไปสู่ความพิการ และการสูญเสียชีวิตได้[1-5] โดยในผู้สูงอายุที่มีอายุมากกว่า 50 ปี มีโอกาสเกิดกระดูกหักจากโรคกระดูกพรุนในช่วงชีวิตที่เหลืออยู่ได้สูงถึง 50% ในเพศหญิง และ 20% ในเพศชาย[6] โรคกระดูกพรุนเป็นโรคที่พบได้บ่อยและมีความชุกค่อนข้างสูง[2,4,6-8] ซึ่งจากการศึกษาของ Reginster JY และคณะคาดการณ์ว่าทั่วโลกมีผู้ป่วยโรคกระดูกพรุนอยู่ถึง 200 ล้านคน[7] นอกจากนี้แล้วยังเป็นโรคที่มีค่าใช้จ่ายในการป้องกันและรักษาสูงเช่นเดียวกัน[4,7,8] โดยการศึกษาของ Burge R และคณะพบว่าในปี ค.ศ. 2005 ประเทศสหรัฐอเมริกามีค่าใช้จ่ายในการรักษาโรคกระดูกพรุนรวมทั้งภาวะกระดูกหักจากโรคกระดูกพรุนอยู่ระหว่าง 13,700-20,300 ล้านดอลลาร์ และจะเพิ่มสูงขึ้นตามจำนวนประชากรผู้สูงอายุที่มากขึ้น และคาดการณ์ว่าในปี ค.ศ. 2025 จะมีค่าใช้จ่ายในการรักษาสูงถึง 25,300 ล้านดอลลาร์[8] สำหรับในประเทศไทยนั้นมีการศึกษาพบอุบัติการณ์ของโรคกระดูกพรุนในผู้หญิงอายุ 40-80 ปี โดยแยกเป็นกระดูกพรุนที่บริเวณกระดูกสะโพก 13.6% และกระดูกพรุนที่บริเวณกระดูกสันหลัง 19.8% ตามลำดับ สำหรับค่าใช้จ่ายนั้นพบว่าผู้ป่วยแต่ละรายจะมีรายจ่ายเพื่อการรักษาโรคกระดูกพรุนอยู่ที่ 36,563 บาทต่อคนต่อปี แต่ถ้าหากมีกระดูกสะโพกหักร่วมด้วยจะมีรายจ่ายเพิ่มสูงขึ้นถึง 118,168 บาทต่อคนต่อปี[4]

สำหรับการรักษาโรคกระดูกพรุนนั้น ในปัจจุบันยากลุ่ม Bisphosphonates ถือเป็นยาหลักที่ใช้ในการรักษา[5,9] จากแนวปฏิบัติบริการสาธารณสุขโรคกระดูกพรุน พ.ศ. 2553 ของราชวิทยาลัยแพทย์ออร์โธปิดิกส์แห่งประเทศไทย ได้ระบุข้อบ่งชี้ของการรักษาโรคกระดูกพรุนด้วยยาในผู้ป่วยหญิงวัยหมดประจำเดือน หรือผู้ป่วยชายที่มีอายุตั้งแต่ 50 ปีขึ้นไปดังต่อไปนี้[9]

1. มีประวัติกระดูกสันหลังหรือกระดูกสะโพกหักอันเนื่องมาจากภยันตรายที่ไม่รุนแรง หรือ

2. ผู้ที่มีความหนาแน่นมวลกระดูก (Bone mineral density [BMD]) จากการตรวจวินิจฉัยด้วยเครื่อง DXA (Dual energy X-ray absorptiometry) scan ที่ตำแหน่งกระดูกคอสะโพก (Femoral neck) หรือตำแหน่งกระดูกสะโพกโดยรวม (Total hip) หรือกระดูกสันหลังส่วนเอวระดับ L1-L4 มีค่า T-score น้อยกว่าหรือเท่ากับ -2.5 หรือ

3. ผู้ที่มีความหนาแน่นมวลกระดูกจากการตรวจวินิจฉัยด้วยเครื่อง DXA scan ที่ตำแหน่งกระดูกคอสะโพก หรือตำแหน่งกระดูกสะโพกโดยรวม หรือกระดูกสันหลังส่วนเอวระดับ L1-L4 ได้ผลอยู่ในเกณฑ์กระดูกบาง (T-score อยู่ระหว่าง -1.0 ถึง -2.5) ร่วมกับมีความเสี่ยงสูงในการเกิดกระดูกหัก โดยประเมินจากการคำนวณโดยใช้คะแนนของ Fracture risk assessment tool (FRAX™) แล้วพบว่าโอกาสเสี่ยงในการเกิดกระดูกสะโพกหักในอีก 10 ปีข้างหน้า (10-year probability of hip fracture) มากกว่าหรือเท่ากับ 3% หรือโอกาสเสี่ยงในการเกิดกระดูกหักในบริเวณอื่นๆ ในอีก 10 ปีข้างหน้า (10-year probability of major osteoporotic fractures) มากกว่าหรือเท่ากับ 20%

สำหรับยา Alendronate ที่เป็นยาในกลุ่ม Bisphosphonates นั้นมีคุณสมบัติทั้งป้องกันและรักษาโรคกระดูกพรุนได้[5,9,10] จากการศึกษาของ Black DM และคณะพบว่ายา Alendronate สามารถเพิ่มความหนาแน่นของมวลกระดูก รวมทั้งลดความเสี่ยงของการเกิดกระดูกหักที่กระดูกสันหลัง กระดูกสะโพก และกระดูกตำแหน่งอื่นนอกเหนือจากกระดูกสันหลังได้อีกด้วย โดยเฉพาะอย่างยิ่งสามารถลดความเสี่ยงของกระดูกสันหลังยุบหรือหักจากโรคกระดูกพรุนได้ถึง 47%[10]

ถึงแม้ว่าการรักษาโรคกระดูกพรุนสามารถทำได้ง่ายโดยการให้ยากลุ่ม Bisphosphonates แต่ปัญหาหลักอันหนึ่งที่สำคัญคือการเข้าถึงการรักษาเนื่องจากยามีราคาสูง โดยเฉพาะอย่างยิ่งยาต้นแบบ (Original หรือ Brand drugs) ที่มีราคาสูงกว่ายาสามัญ (Local หรือ Generic drugs) ประมาณ 2-3 เท่า สำหรับประเทศไทยในปัจจุบันนั้นได้มีการนำเข้ายา Alendronate สามัญ คือ Bonmax® (บริษัท Apotex Incorporated ประเทศแคนาดา) ซึ่งเป็นยาที่คาดว่าสามารถนำมาใช้ทดแทนยา Alendronate ต้นแบบได้ อย่างไรก็ตามยังไม่เคยมีการศึกษาเปรียบเทียบประสิทธิภาพของยา Bonmax® กับยา Alendronate ต้นแบบมาก่อน ดังนั้นการศึกษานี้จึงมีความสำคัญหากพบว่ายา Alendronate สามัญมีประสิทธิภาพในการรักษาโรคกระดูกพรุนได้ไม่ต่างจากยาต้นแบบจะทำให้ประชาชนสามารถเข้าถึงการรักษาได้ง่ายขึ้น ซึ่งจะเกิดประโยชน์อย่างมากต่อวงการสาธารณสุขของประเทศไทย

8.2 **วัตถุประสงค์ของการวิจัย** (Objective)

8.2.1 **วัตถุประสงค์หลัก** (Primary outcome)

เพื่อศึกษาเปรียบเทียบการเปลี่ยนแปลงของมวลกระดูกบริเวณกระดูกสันหลังระดับ L1-L4 ที่ระยะเวลา 1 ปี ในผู้ป่วยโรคกระดูกพรุนที่ได้รับการรักษาด้วยยา Alendronate ต้นแบบหรือสามัญ

8.2.2 **วัตถุประสงค์รอง** (Secondary outcome)

1) เพื่อศึกษาเปรียบเทียบการเปลี่ยนแปลงของมวลกระดูกบริเวณกระดูกคอสะโพก และกระดูกสะโพกโดยรวม ที่ระยะเวลา 1 ปี ในผู้ป่วยโรคกระดูกพรุนที่ได้รับการรักษาด้วยยา Alendronate ต้นแบบหรือสามัญ

2) เพื่อศึกษาเปรียบเทียบการเปลี่ยนแปลงของ Bone markers (β-CTX และ P1NP) ที่ระยะเวลา 3, 6 และ 12 เดือน ในผู้ป่วยโรคกระดูกพรุนที่ได้รับการรักษาด้วยยา Alendronate ต้นแบบหรือสามัญ

3) เพื่อศึกษาเปรียบเทียบความปลอดภัยและผลข้างเคียงของการใช้ยา Alendronate ต้นแบบและสามัญในผู้ป่วยโรคกระดูกพรุน

4) เพื่อศึกษาเปรียบเทียบคะแนนคุณภาพชีวิตจากแบบประเมิน EQ-5D™ ฉบับภาษาไทย ที่ระยะเวลา 1 ปี ในผู้ป่วยโรคกระดูกพรุนที่ได้รับการรักษาด้วยยา Alendronate ต้นแบบหรือสามัญ

8.3 **ประเภทของโครงการวิจัย** (Research type)

☑ Experimental biomedical/Clinical research

- ❏ Drug trial ระบุชื่อยา
  - ❏ Registered drug
  - ❏ Investigational (new) drug
- ❏ Medical device trial ระบุชื่อเครื่องมือ
  - ❏ Registered device
  - ❏ Investigational (new) device
- ❏ Vaccine trial ระบุชื่อ/รหัสวัคซีน
  - ❏ Registered vaccine
  - ❏ Investigational (new) vaccine
- ☑ Experimental procedure/intervention ระบุ การให้ยา Alendronate ต้นแบบ (Fosamax®) หรือสามัญ (Bonmax®)
  - ❏ High risk ☑ Minimal risk
- ❏ Bioequivalence
- ❏ *In vitro*/laboratory-based study
- ❏ Research using repository of biological products (cells, blood, tissues, fluids, etc.)
- ❏ อื่นๆ ระบุ

❏ Observation clinical research

- ❏ Prospective (cohort) study
- ❏ Case series
- ❏ Retrospective (chart) review
- ❏ Epidemiology research
  - ❏ Surveillance
  - ❏ Monitoring

❑ อื่นๆ ระบุ

❑ Social/Behavioral research

❑ Questionnaire-based research

❑ อื่นๆ ระบุ

8.4 **การออกแบบการวิจัย** (Research design)

☑ Randomized-controlled trial

❑ Quasi-experimental study (manipulation and control only, without randomization)

❑ Pre-experimental study (manipulation only, without control and randomization)

❑ Prospective cohort study

❑ Descriptive study

❑ Cross-sectional study

❑ Pilot study

❑ อื่นๆ ระบุ

8.5 **ผู้ร่วมวิจัย/อาสาสมัคร** (Research subjects)

การคำนวณขนาดตัวอย่าง (Sample size calculation)

สูตรการคำนวณขนาดตัวอย่างเพื่อการเปรียบเทียบค่าเฉลี่ยของกลุ่มประชากร โดยกำหนดสมมติฐานการวิจัยที่ว่า ยา Alendronate สามัญมีประสิทธิภาพในการรักษาโรคกระดูกพรุนไม่ด้อยไปกว่ายา Alendronate ต้นแบบ (Non-inferiority test)[11,12] มีดังนี้

$$n = \frac{(Z_\alpha + Z_\beta)^2(\sigma_0^2 + \sigma_1^2)}{\delta^2}$$

โดยกำหนดค่า Z score สำหรับโอกาสที่จะเกิด Error ชนิดต่างๆ โดยกำหนดให้มีนัยสำคัญทางสถิติ (Statistical significance) เมื่อ Type I error (α) เท่ากับ 0.05 และ Type II error (β) เท่ากับ 0.2 ดังนั้น Z_α ($Z_{0.05}$) จึงมีค่าเท่ากับ 1.645 และ Z_β ($Z_{0.2}$) มีค่าเท่ากับ 0.842 ตามลำดับ สำหรับค่าส่วนเบี่ยงเบนมาตรฐาน (Standard deviation) ของมวลกระดูก (BMD) บริเวณกระดูกสันหลังในผู้ป่วยโรคกระดูกพรุนที่ได้รับยา Alendronate จากการศึกษาของ Grima DT และคณะ[13] พบว่ามีค่าส่วนเบี่ยงเบนมาตรฐานของผู้ป่วยที่ได้รับยา Alendronate ต้นแบบเท่ากับ 0.138 ส่วนค่าขอบเขตความแตกต่างที่ยอมรับได้ (Non-inferiority margin) ระหว่างค่าเฉลี่ยของมวลกระดูก (BMD) ของกลุ่มที่ได้รับยาต้นแบบและกลุ่มที่ได้รับยาสามัญนั้นกำหนดให้เป็น 0.5 เท่าของส่วนเบี่ยงเบนมาตรฐาน (σ) ซึ่งเมื่อแทนค่าต่างๆ ลงในสูตรแล้ว จะได้ขนาดตัวอย่างต่อกลุ่มที่ควรใช้ในแต่ละกลุ่มคือ 50 ราย

อย่างไรก็ตาม เนื่องจากโครงการวิจัยนี้ใช้ระยะเวลาในการติดตามรักษาเป็นเวลานาน อาจมีผู้ป่วยบางรายไม่ให้ความร่วมมือในการประเมิน (Poor compliance) หรือไม่มาตรวจติดตาม (Loss follow up) ได้ ซึ่งคาดว่าจะมีผู้ป่วยดังกล่าวประมาณ

20% (10 ราย) ดังนั้นผู้วิจัยจึงคัดเลือกผู้ร่วมวิจัย/อาสาสมัครในแต่ละกลุ่มเป็นกลุ่มละ 60 ราย รวมผู้ร่วมวิจัย/อาสาสมัครทั้ง 2 กลุ่มเป็นทั้งหมด 120 ราย

เกณฑ์การคัดเลือกผู้ร่วมวิจัย/อาสาสมัคร (Inclusion criteria)

ผู้ป่วยหญิงวัยหมดประจำเดือนหรือผู้ป่วยชายที่มีอายุมากกว่า 50 ปีและมีข้อบ่งชี้ของการใช้ยาในการรักษาโรคกระดูกพรุนตามแนวปฏิบัติบริการสาธารณสุขโรคกระดูกพรุน พ.ศ. 2553 ของราชวิทยาลัยแพทย์ออร์โธปิดิกส์แห่งประเทศไทย[9] ดังนี้

1) มีประวัติกระดูกสันหลังหักยุบหรือกระดูกสะโพกหักอันเนื่องมาจากภยันตรายที่ไม่รุนแรง โดยจะทำการคัดเลือกผู้ป่วยที่ผ่านการรักษาเรื่องกระดูกหักมาแล้วอย่างน้อย 3 เดือน หรือ
2) ตรวจวัดความหนาแน่นมวลกระดูก (Bone mineral density [BMD]) จากเครื่อง DXA scan ที่ตำแหน่งกระดูกคอสะโพก (Femoral neck) หรือตำแหน่งกระดูกสะโพกโดยรวม (Total hip) หรือกระดูกสันหลังส่วนเอวระดับ L1-L4 แล้วมีค่า T-score น้อยกว่าหรือเท่ากับ -2.5 หรือ
3) ตรวจวัดความหนาแน่นมวลกระดูกด้วยเครื่อง DXA scan ที่ตำแหน่งกระดูกคอสะโพก หรือตำแหน่งกระดูกสะโพกโดยรวม หรือกระดูกสันหลังส่วนเอวระดับ L1-L4 แล้วได้ผลอยู่ในเกณฑ์กระดูกบาง (T-score อยู่ระหว่าง -1.0 ถึง -2.5) ร่วมกับการประเมินความเสี่ยงของการเกิดกระดูกหักโดยใช้ FRAX™ แล้วพบว่าโอกาสเสี่ยงในการเกิดกระดูกสะโพกหักในอีก 10 ปีข้างหน้า มากกว่าหรือเท่ากับ 3% หรือโอกาสเสี่ยงในการเกิดกระดูกหักในบริเวณอื่นๆ ในอีก 10 ปีข้างหน้า มากกว่าหรือเท่ากับ 20%

เกณฑ์การคัดออกผู้ร่วมวิจัย/อาสาสมัคร (Exclusion criteria)

1) ผู้ป่วยที่มีข้อห้ามหรือผู้ที่อาจจะเกิดภาวะแทรกซ้อนจากการใช้ยาในกลุ่ม Bisphosphonates ได้ เช่น มีอาการแพ้ยาในกลุ่ม Bisphosphonates หรือเป็นโรคหลอดอาหารอักเสบ หรือโรคกรดไหลย้อน เป็นต้น
2) ผู้ป่วยที่มีระดับแคลเซียมในเลือดผิดปกติ (มากกว่า 10.2 mg/dl หรือน้อยกว่า 8.5 mg/dl)
3) ผู้ป่วยที่มีการทำงานของไตบกพร่อง โดยวัดจากอัตราการกรองของหน่วยไต (GFR) น้อยกว่า 35 ml/min/1.73 m^2
4) ผู้ป่วยมีภาวะอื่นๆ ที่มีผลกระทบต่อกระบวนการ Bone metabolism เช่น ภาวะ Hyperparathyroidism, Paget's disease, Renal osteodystrophy, Rheumatoid arthritis, severe vitamin D deficiency (serum 25-hydroxyvitamin D < 10 ng/mL) เป็นต้น
5) ผู้ป่วยที่ได้รับการรักษาโรคกระดูกพรุนด้วยยาในกลุ่ม Bisphosphonates หรือ Calcitonin หรือ Teriperatide ภายในระยะเวลา 1 ปี
6) ผู้ป่วยที่ได้รับยาในกลุ่ม Glucocorticoids ขนาดมากกว่า 5 mg/วัน ภายในระยะเวลา 6 เดือน

เกณฑ์การถอนผู้ร่วมวิจัย/อาสาสมัครหรือยุติการเข้าร่วมการวิจัย (Withdrawal or termination criteria)

1) ผู้ร่วมวิจัย/อาสาสมัครเกิดภาวะแทรกซ้อนรุนแรงจากการรับประทานยาที่ใช้ในการวิจัย เช่นเกิดภาวะแพ้ยา มีอาการปวดท้องอย่างรุนแรงภายหลังการรับประทานยา หรือมีกระดูกหักใหม่ซึ่งแพทย์ผู้ทำการรักษามีความกังวลต่อผลของการติดของกระดูกภายหลังจากได้รับยา Bisphosphonate
2) ผู้ร่วมวิจัย/อาสาสมัครมีความประสงค์จะออกจากการโครงการวิจัย

3) เมื่อทำการวิเคราะห์ระหว่างดำเนินการ (Interim analysis) ในแง่ของความเสี่ยงของทั้งโครงการแล้วพบว่าการใช้ยา Alendronate สามัญทำให้เกิดผลเสียต่อผู้ร่วมวิจัย/อาสาสมัครมากกว่าการใช้ยา Alendronate ต้นแบบอย่างชัดเจน

8.6 กระบวนการวิจัย

กระบวนการวิจัยมีขั้นตอนต่างๆ โดยเริ่มจากคัดกรองผู้หญิงวัยหมดประจำเดือนหรือผู้ชายที่มีอายุมากกว่า 50 ปี และอยู่ในเกณฑ์การคัดเลือกผู้ร่วมวิจัย/อาสาสมัคร (Inclusion criteria) รวมทั้งไม่อยู่ในกลุ่มของผู้ที่มีคุณสมบัติการคัดออกผู้ร่วมวิจัย/อาสาสมัคร (Exclusion criteria) ในกรณีที่มีกระดูกหักมาก่อน ผู้ร่วมวิจัย/อาสาสมัครจะต้องผ่านการรักษาเรื่องกระดูกหักมาแล้วอย่างน้อย 3 เดือน หลังจากผ่านขั้นตอนการคัดกรองแล้ว ผู้ร่วมวิจัย/อาสาสมัครจะได้รับการอธิบายเกี่ยวกับลักษณะของโครงการวิจัย ตั้งแต่ความสำคัญ ข้อดี ข้อเสีย ประโยชน์ที่จะได้รับ รวมทั้งวิธีการและกระบวนการวิจัย ระยะเวลาในการเข้าร่วมวิจัย การประเมินผล ตลอดจนผลข้างเคียงที่อาจจะเกิดขึ้นได้ระหว่างการเข้าร่วมงานวิจัยนี้ หากผู้ร่วมวิจัย/อาสาสมัครยินดีที่จะเข้าร่วมโครงการวิจัยให้ลงชื่อในหนังสือแสดงเจตนายินยอมเข้าร่วมการวิจัย (Consent form) และเอกสารชี้แจงผู้ร่วมวิจัย/อาสาสมัคร (Participant information sheet) ก่อนจัดกลุ่มผู้ร่วมวิจัย/อาสาสมัครเพื่อเริ่มกระบวนการวิจัยต่อไป จากนั้นผู้ร่วมวิจัย/อาสาสมัครจะถูกแบ่งออกเป็น 2 กลุ่มโดยวิธีการสุ่มตัวอย่าง (Block randomization) จากคอมพิวเตอร์ (Computer generated list) โดยมีรายละเอียดในการให้ยาดังนี้ โดยกลุ่มที่ 1 (Generic alendronate group) ผู้ร่วมวิจัย/อาสาสมัครจะได้รับยา Alendronate สามัญ (Bonmax®) ขนาด 70 มิลลิกรัม ให้รับประทานสัปดาห์ละ 1 เม็ด ก่อนอาหารเช้า โดยให้รับประทานติดต่อกันเป็นระยะเวลา 1 ปี ส่วนกลุ่มที่ 2 (Brand alendronate group) ผู้ร่วมวิจัย/อาสาสมัครได้รับยา Alendronate ต้นแบบ (Fosamax®) ขนาด 70 มิลลิกรัม ให้รับประทานสัปดาห์ละ 1 เม็ด ก่อนอาหารเช้า โดยให้รับประทานติดต่อกันเป็นระยะเวลา 1 ปี

ผู้ร่วมวิจัย/อาสาสมัครจะได้รับการตรวจมวลกระดูก ทำแบบประเมินคุณภาพชีวิต (EQ-5D™ ฉบับภาษาไทย) และเจาะเลือดเพื่อใช้เป็นข้อมูลในการวิเคราะห์ผลการวิจัย โดยการเจาะเลือดจะทำทั้งหมด 4 ครั้ง ครั้งละ 10 มิลลิลิตร รวมทั้ง 4 ครั้งเป็นปริมาณ 40 มิลลิลิตร โดยครั้งแรกจะทำในวันที่เข้าร่วมการวิจัย ครั้งที่ 2 หลังเข้าร่วมวิจัย 3 เดือน ครั้งที่ 3 หลังเข้าร่วมวิจัย 6 เดือน และครั้งสุดท้ายที่หลังเข้าร่วมวิจัย 1 ปี ซึ่งรายละเอียดการเก็บข้อมูลผลเลือด การตรวจมวลกระดูก และคะแนนจากแบบประเมินคุณภาพชีวิตจะนำไปบันทึกตามตาราง Case record form ดังที่แนบมาในส่วนท้ายของแบบโครงร่างวิจัยนี้

ผู้ร่วมวิจัย/อาสาสมัครทุกรายจะได้รับแคลเซียมเสริมในรูปแบบของ Calcium carbonate ($CaCO_3$) โดยให้รับประทานขนาด 1,000 mg/วัน ร่วมกับวิตามินดีในขนาดที่เหมาะสมเพื่อรักษาระดับวิตามินดีในเลือด [25(OH)D] ให้มีค่ามากกว่าหรือเท่ากับ 30 ng/mL สำหรับการเก็บข้อมูลเพื่อวิเคราะห์ นอกจากการเจาะเลือด ผู้ร่วมวิจัย/อาสาสมัครจะได้รับการประเมินประสิทธิภาพของยาโดยการตรวจมวลกระดูกด้วยเครื่อง DXA scan รวมทั้งประเมินคุณภาพชีวิตจากแบบประเมิน EQ-5D™ ฉบับภาษาไทยทั้งหมด 2 ครั้ง ครั้งแรกในวันที่เข้าร่วมการวิจัย และครั้งที่ 2 หลังเข้าร่วมวิจัย 1 ปี โดยการตรวจมวลกระดูก และการประเมินคุณภาพชีวิตจะทำในวันเดียวกันกับการเจาะเลือดเพื่อความสะดวกของผู้ป่วย สำหรับการพบแพทย์นั้น ผู้ร่วมวิจัย/อาสาสมัครจะต้องมาพบแพทย์จำนวน 4 ครั้งเพื่อตรวจติดตามการรักษาและประเมินประสิทธิภาพของยา รวมทั้งสอบถามอาการข้างเคียงจากการใช้ยาที่อาจเกิดขึ้นได้ โดยแต่ละครั้งนั้นจะใช้เวลาประมาณ 15 นาที การพบแพทย์ครั้งแรกจะทำในวันที่เข้าร่วมการวิจัย หลังจากนั้นจะได้รับการนัดหมายเพิ่มเติมอีก 3 ครั้ง คือ 3 เดือน 6 เดือน และ 12 เดือนหลังจากที่ได้พบแพทย์ครั้งแรก

8.7 กระบวนการเก็บข้อมูล (Data collection process)

ผู้วิจัยจะเก็บข้อมูลของผู้ป่วยดังต่อไปนี้ลงในแบบบันทึกการเก็บข้อมูล (Case record form)

1) วันที่เข้าร่วมงานวิจัย

2) ข้อมูลส่วนบุคคล ได้แก่ เพศ อายุ ภูมิลำเนา อาชีพ การศึกษา น้ำหนัก ส่วนสูง โรคประจำตัว และยาที่รับประทานเป็นประจำ

3) ข้อบ่งชี้ในการได้รับยารักษาโรคกระดูกพรุน

4) ประวัติการเข้ารับการผ่าตัดมาก่อนหน้านี้

5) ประวัติกระดูกหักมาก่อนหน้านี้

6) ผลการตรวจเลือดดังต่อไปนี้ Hemoglobin (Hb), Hematocrit (Hct), BUN, Creatinine (Cr), AST, ALT, Alkaline phosphatase (ALP), Vitamin D level [25(OH)D], Total calcium, Phosphate, Parathyroid hormone (PTH), Bone markers (β-CTX และ P1NP)

7) ผลการตรวจมวลกระดูก

8) ผลข้างเคียงจากการใช้ยา ได้แก่ ปวดท้อง คลื่นไส้/อาเจียน แสบร้อนหน้าอก ท้องอืด ท้องผูก ท้องเสีย ผื่นแพ้ ปวดเมื่อยกล้ามเนื้อ และผลข้างเคียงอื่นๆ

9) แบบประเมินคุณภาพชีวิต (EQ-5D™ ฉบับภาษาไทย) ซึ่งแบบประเมินนี้เป็นแบบสอบถามคุณภาพชีวิตด้านสุขภาพ โดยผู้ร่วมวิจัย/อาสาสมัครจะเป็นผู้ทำแบบสอบถามด้วยตนเอง จากการศึกษาของ Kimman M และคณะพบว่าแบบทดสอบ EQ-5D™ ฉบับภาษาไทยสามารถนำมาประเมินคุณภาพชีวิตในประชากรไทยโดยทั่วไปได้[14] สำหรับแบบทดสอบนี้จะประกอบด้วย 2 ส่วน คือ ส่วนที่ 1 เป็นคำถามที่เกี่ยวข้องกับสุขภาพและการดำเนินชีวิต มีทั้งหมด 5 ข้อ ได้แก่ ความสามารถในการเคลื่อนไหว (Mobility) การดูแลตนเอง (Self-care) กิจกรรมที่ทำเป็นประจำ (Usual activities) อาการเจ็บปวดหรือไม่สบายตัว (Pain/discomfort) และความวิตกกังวลหรือความซึมเศร้า (Anxiety/depression) โดยแต่ละคำถามจะมีคำตอบให้เลือก 5 ระดับ ในส่วนที่ 2 จะเป็นมาตรวัดสภาวะสุขภาพทางตรง หรือ Visual analog scale (VAS) ซึ่งมีลักษณะเป็นเส้นตรงและแบ่งช่องคะแนนตั้งแต่ 0 ถึง 100 คะแนน โดย 0 คะแนนหมายถึงสุขภาพที่แย่สุด และ 100 คะแนนหมายถึงสุขภาพที่ดีที่สุด

8.8 การวัดผล/การวิเคราะห์ผลการวิจัย (Outcome measurement/Data analysis)

ผลลัพธ์หลัก (Primary outcome) ของการศึกษานี้คือ การเปลี่ยนแปลงของมวลกระดูกบริเวณกระดูกสันหลังระดับ L1-L4 ที่ระยะเวลา 1 ปี ในผู้ป่วยโรคกระดูกพรุนที่ได้รับการรักษาด้วยยา Alendronate ต้นแบบหรือสามัญซึ่งจะมีการรายงานผลเป็นค่า mean $\pm$ SD

ผลลัพธ์รอง (Secondary outcome) ของการศึกษานี้คือ การเปลี่ยนแปลงของมวลกระดูกบริเวณคอกระดูกสะโพก และกระดูกสะโพกโดยรวมที่ระยะเวลา 1 ปี รวมถึงการเปลี่ยนแปลงของ Bone markers (β-CTX และ P1NP) ที่ระยะเวลา 3, 6 และ 12 เดือน ในผู้ป่วยโรคกระดูกพรุนที่ได้รับการรักษาด้วยยา Alendronate ต้นแบบหรือสามัญ รวมทั้งเปรียบเทียบความปลอดภัยและผลข้างเคียงของการใช้ยา และคะแนนประเมินคุณภาพชีวิต ซึ่งทั้งหมดจะมีการรายงานผลเป็นค่า mean $\pm$ SD

ค่าความแตกต่างของมวลกระดูก และ Bone markers ที่ได้ จะนำมาเปรียบเทียบกันระหว่าง 2 กลุ่ม โดยการใช้สถิติชนิด Unpaired t-test สำหรับความแตกต่างของมวลกระดูก และ Repeated measures ANOVA สำหรับความแตกต่างของ Bone markers เพื่อหาความสัมพันธ์ของข้อมูลแบบต่อเนื่อง (Continuous data) ระหว่างกลุ่มที่ได้ยา Alendronate ต้นแบบและ

สามัญ สำหรับข้อมูลส่วนบุคคล (Demographic data) ที่เป็นข้อมูลชนิด Categorial data ผู้วิจัยจะใช้สถิติชนิด Chi-square test เพื่อเปรียบเทียบและหาความสัมพันธ์ของข้อมูลระหว่างกลุ่มตัวอย่าง 2 กลุ่ม

8.9 หลักฐาน ข้อมูล หรือเอกสารอ้างอิง (References)

1) Brown JP, Davison KS, Olszynski WP, Beattie KA, Adachi JD. A critical review of brand and generic alendronate for the treatment of osteoporosis. SpringerPlus 2013;2:550.
2) Ringe JD, Möller G. Differences in persistence, safety and efficacy of generic and original branded once weekly bisphosphonates in patients with postmenopausal osteoporosis: 1-year results of a retrospective patient chart review analysis. Rheumatol Int 2009;30:213-221.
3) Kanis JA, Reginster JY, Kaufman JM, Ringe JD, Adachi JD, Hiligsmann M, Rizzoli R, Cooper C. Reappraisal of generic bisphosphonates in osteoporosis. Osteoporos Int 2012;23:213-221.
4) Pongchaiyakul C, Songpattanasilp T, Taechakraichana N. Burden of osteoporosis in Thailand. J Med Assoc Thai 2008;91(2):261-267.
5) National Osteoporosis Foundation. Clinician's guide to prevention and treatment of osteoporosis. Washington, DC: National Osteoporosis Foundation; 2013.
6) van den Bergh JPW, Bouts ME, van der Veer E, van der Velde RY, Janssen MJW, Geusens PP, Winkens B, Oldenhof NJJ, van Geel TACM. Comparing tolerability and efficacy of generic versus brand alendronate: A randomized clinical study in postmenopausal women with a recent fracture. Plos One 2013;8:10.
7) Reginster JV, Burlet N. Osteoporosis: A still increasing prevalence. Bone 2006;38:S4-S9.
8) Burge R, Dawson-Hughes B, Solomon DH, Wong JB, King A, Tosteson A. Incidence and economic burden of osteoporosis-Related fractures in the United States, 2005-2025. J Bone Miner Res 2007;22(3):465-475.
9) ราชวิทยาลัยแพทย์ออร์โธปิดิกส์แห่งประเทศไทยและมูลนิธิโรคกระดูกพรุนแห่งประเทศไทย. แนวปฏิบัติบริการสาธารณสุขโรคกระดูกพรุน พ.ศ. 2553. กรุงเทพฯ: ราชวิทยาลัยแพทย์ออร์โธปิดิกส์แห่งประเทศไทย; 2553.
10) Black DM, Cummings SR, Karpf DB, Cauley JA, Thompson DE, Nevitt MC, Bauer DC, Genant HK, Haskell WL, Marcus R, Ott SM, Torner JC, Quandt SA, Reiss TF, Ensrud KE. Randomised trial of effect of alendronate on risk of fracture in women with existing vertebral fractures. Lancet 1996;348:1535-1541.
11) Chow SC, Shao J, Wang H. Sample size calculations in clinical research. Basel: Marcel Dekker, Inc.;2003.
12) Hayes RJ, Benett S. Simple sample size calculation for cluster-randomized trials. Int J Epidemiol 1999;28:319-326.
13) Grima DT, Papaioannou A, Airia P, Ioannidis G, Adachi JD. Adverse events, bone mineral density and discontinuation associated with generic alendronate among postmenopausal women previously tolerant of brand alendronate: a retrospective cohort study. BMC Musculoskelet Disord 2010;11:68.
14) Kimman M, Vathesatogkit P, Woodward M, E ST, Thumboo J, Sukit Yamwong S, Ratanachaiwong W, Hwee LW, Sritara P. Validity of the Thai EQ-5D in an occupational population in Thailand. Qual Life Res 2013;22:1499–1506.

ข้อพิจารณาด้านจริยธรรมการวิจัยในคน (Ethical consideration)

9. **ลักษณะผู้ร่วมวิจัย/อาสาสมัคร**

❑ Healthy volunteers

☑ Patients excluding vulnerable subjects

❑ อื่นๆ เช่น Retrospective chart review

❑ Vulnerable subjects ระบุ

10. **การใช้ข้อมูลและการเก็บชีววัตถุของผู้ร่วมวิจัย/อาสาสมัคร**

☑ ไม่มี

❑ มี ระบุ

11. **กระบวนการเชิญชวนให้เข้าร่วมการวิจัย** (Recruitment process)

11.1 **สถานที่**

คลินิกโรคกระดูกเมตะบอลิก (Metabolic bone disease (MBD) clinic) หน่วยตรวจและติดตามผลการรักษาออร์โธปิดิคส์ฯ (ห้องเฝือก) อาคารสยามินทร์ โรงพยาบาลศิริราช

11.2 **กระบวนการ**

ผู้ร่วมวิจัย/อาสาสมัครจะได้รับการอธิบายเกี่ยวกับลักษณะของโครงการวิจัย ตั้งแต่ความสำคัญ ข้อดี ข้อเสีย ประโยชน์ที่จะได้รับ รวมทั้งวิธีการและกระบวนการวิจัย ระยะเวลาในการเข้าร่วมวิจัย การประเมินผล ตลอดจนผลข้างเคียงที่อาจจะเกิดขึ้นได้ระหว่างการเข้าร่วมงานวิจัยนี้ พร้อมทั้งให้เอกสารชี้แจงเกี่ยวกับงานวิจัยไปศึกษาก่อนการตัดสินใจเข้าร่วมการวิจัย โดยผู้วิจัยร่วมจะทำการเชิญชวนผู้ป่วยเพื่อเข้าร่วมการวิจัยระหว่างมารับการตรวจติดตามที่โรงพยาบาล หากผู้ป่วยยินดีเข้าร่วมการวิจัยจะต้องลงนามยินยอมเข้าร่วมงานวิจัยด้วยความสมัครใจก่อนทุกครั้ง

12. **กระบวนการขอความยินยอมให้เข้าร่วมการวิจัย** (Informed consent process)

❑ ไม่เกี่ยวข้องเนื่องจากเป็น Retrospective chart review

❑ ขอยกเว้นกระบวนขอความยินยอม

☑ ขอความยินยอมให้ระบุข้อมูลต่อไปนี้ (Need inform consent)

ผู้ร่วมวิจัย/อาสาสมัครจะได้รับการอธิบายเกี่ยวกับลักษณะของโครงการวิจัย ตั้งแต่ความสำคัญ ข้อดี ข้อเสีย ประโยชน์ที่จะได้รับ รวมทั้งวิธีการและกระบวนการวิจัย ระยะเวลาในการเข้าร่วมวิจัย การประเมินผล ตลอดจนผลข้างเคียงที่อาจจะเกิดขึ้นได้ระหว่างการเข้าร่วมงานวิจัยนี้ พร้อมทั้งให้เอกสารชี้แจงเกี่ยวกับงานวิจัยไปศึกษาก่อนการตัดสินใจเข้าร่วมการวิจัย โดยผู้วิจัยร่วมจะทำการเชิญชวนผู้ป่วยเพื่อเข้าร่วมการวิจัยระหว่างมารับการตรวจติดตามที่โรงพยาบาล หากผู้ป่วยยินดีเข้าร่วมการวิจัยจะต้องลงนามยินยอมเข้าร่วมงานวิจัยด้วยความสมัครใจก่อนทุกครั้ง

13. **ประโยชน์ที่คาดว่าจะได้รับจากการวิจัย**

ประโยชน์ในการเข้าถึงการรักษาโรคกระดูกพรุนซึ่งเป็นภาวะที่พบได้บ่อย และเข้าถึงการรักษาได้น้อย จึงเป็นประโยชน์โดยตรงต่อผู้ร่วมวิจัย/อาสาสมัครในการเข้าถึงการรักษา เมื่อผู้ป่วยโรคกระดูกพรุนได้รับการรักษาเร็ว ทำให้มีผลป้องกัน และลดการเกิดภาวะแทรกซ้อนจากภาวะกระดูกหักจากโรคกระดูกพรุนได้ ซึ่งเป็นการช่วยลดภาระค่าใช้จ่ายที่ประเทศชาติต้องสูญเสียในแต่ละปีไปได้มาก รวมทั้งทำให้ประชาชนโดยทั่วไปมีคุณภาพชีวิตที่ดีขึ้น นอกจากนี้หากการวิจัยพบว่า ยา Alendronate สามัญมีประสิทธิภาพและความปลอดภัยไม่ต่างจากยาต้นแบบแล้ว ผู้ป่วยโรคกระดูกพรุนจะสามารถเข้าถึงยาได้มากกว่า เนื่องจากมีราคาที่ต่ำกว่ายาต้นแบบถึง 2-3 เท่า

14. **ผลกระทบที่อาจจะเกิดแก่ผู้ร่วมวิจัย/อาสาสมัคร**

ผลข้างเคียงจากการใช้ยา Alendronate โดยปกติมักพบอาการที่เป็นผลข้างเคียงหลังการรับประทานยาในกลุ่ม Bisphosphonates ได้ค่อนข้างน้อย โดยอาการที่พบได้ คือ การระคายเคืองต่อเยื่อบุหลอดอาหาร อาจมีหลอดอาหารอักเสบ (Esophagitis) ทำให้มีอาการแสบร้อนบริเวณหน้าอก ปวดท้อง แน่นหน้าอก หรือคลื่นไส้ได้ นอกจากนี้ ยังอาจพบผลข้างเคียงอื่นๆ ได้ เช่น ปวดเมื่อยกล้ามเนื้อ ปวดกระดูก อ่อนเพลีย ปวดศีรษะ หรือมีไข้หลังรับประทานยา รวมทั้งอาจเกิดผื่นลมพิษ (Urticarial rash) หลอดเลือดอักเสบที่ผิวหนัง (Cutaneous vasculitis) หรือการบวมใต้ผิวหนังและเยื่อเมือก (Angioedema) จากการแพ้ยา ซึ่งพบได้น้อยเช่นกัน ภาวะอื่นๆ ที่อาจพบได้ ได้แก่ ภาวะแคลเซียมในเลือดต่ำ ซึ่งส่วนใหญ่มักไม่ก่อให้เกิดอาการหรืออาการแสดงผิดปกติทางคลินิก ภาวะกระดูกขากรรไกรตาย (Osteonecrosis of the jaw) และ กระดูกต้นขาหักชนิดพิเศษ (Atypical femoral fracture) ซึ่งภาวะทั้งสองนี้มักพบในผู้ป่วยที่ได้รับยา Bisphosphonates ติดต่อกันเป็นระยะเวลานาน (โดยทั่วไปได้รับยาติดต่อกันมากกว่า 5 ปี) สำหรับงานวิจัยนี้ผู้ร่วมวิจัย/อาสาสมัคร จะต้องไม่เคยได้รับยา Bisphosphonates มาก่อนภายในระยะเวลา 1 ปีที่ผ่านมา ดังนั้นจึงไม่น่าพบภาวะแทรกซ้อนดังกล่าวจากการศึกษานี้

15. **เกี่ยวข้องหรืออาจมีผลกระทบกระเทือนต่อศาสนา ความเชื่อ ขนบธรรมเนียมประเพณีหรือวัฒนธรรมอันดีงาม ชื่อเสียงของสถาบัน ท้องถิ่นหรือประเทศที่ทำการวิจัยอยู่หรือไม่**

❑ เกี่ยวข้อง ระบุวิธีการป้องกันหรือลดผลกระทบดังกล่าว
☑ ไม่เกี่ยวข้อง

16. **วิธีการปกป้องความลับหรือข้อมูลส่วนตัวของผู้ร่วมวิจัย/อาสาสมัคร**

❑ ไม่มีการบันทึกข้อมูลส่วนตัวของผู้ร่วมวิจัย/อาสาสมัคร
☑ มีการบันทึกข้อมูลส่วนตัวของผู้ร่วมวิจัย/อาสาสมัคร

หากมีการบันทึกข้อมูลส่วนตัวดังกล่าวข้างต้น โปรดระบุวิธีการป้องกันบุคคลที่ไม่เกี่ยวข้องในการเข้าถึงข้อมูล ระบุระยะเวลาในการเก็บข้อมูลไว้ และวิธีการทำลายข้อมูลเมื่อสิ้นสุดการวิจัย บันทึกไว้ในคอมพิวเตอร์ส่วนตัวที่มีรหัสป้องกันบุคคลอื่นไม่ให้สามารถเปิดได้โดยเก็บไฟล์ไว้ต่อเป็นเวลา 5 ปี หลังสิ้นสุดการวิจัย

Case no.______________

Date of enrollment______________

Case record form

Demographic data

Gender ❏ Male ❏ Female

*Age*_________________ years old

Hometown ❏ Bangkok and vicinity ❏ Northern ❏ North-eastern

❏ Central ❏ Eastern ❏ Southern

Occupation ❏ None/Housewives ❏ Government officers ❏ Agriculture/Farmers

❏ Private company officers ❏ Students ❏ Others________________________

Educational status ❏ None ❏ Primary school

❏ High school ❏ University/College

*Body weight*___________________ kg *Height*____________________ cm

*BMI*___________________ kg/m^2

Underlying diseases ❏ No

❏ DM ❏ HT ❏ Dyslipidemia ❏ CKD ❏ Liver diseases

❏ Asthma/COPD ❏ Heart diseases/CAD ❏ Stroke/CVA

❏ Others____

*Current medications*____

____

Case no.______________

Previous surgical history ☐ Yes____ ☐ No

Previous fracture history ☐ Yes____ ☐ No

Indication for osteoporosis treatment: Postmenopausal women or men age 50 and older with

☐ History of spinal or hip fractures with low energy trauma

☐ BMD by DXA scan with T-score ≤ -2.5 at the femoral neck, total hip or L1-L4 spine

☐ BMD by DXA scan with T-score between -1 and -2.5 at the femoral neck, total hip or L1-L4 spine and a 10-year hip fracture probability ≥ 3% or a 10-year major osteoporosis-related fracture probability ≥ 20% based on FRAX™

Laboratory and clinical results

Time / Lab		Baseline<br>Date___________	3rd month<br>Date___________	6th month<br>Date___________	12th month<br>Date___________
Hb/Hct					
BUN/Cr					
GFR					
Calcium					
Phosphate					
AST					
ALT					
ALP					
PTH					
25(OH)D					
CTX					
P1NP					
BMD	L1-L4				
	Femoral neck				
	Total hip				
EQ-5D™					

Case no.______________

Complications/Side effects

Complications	3rd month<br>Date_____________	6th month<br>Date______________	12th month<br>Date______________
Stomachache/reflux			
Nausea/vomiting			
Bloating			
Diarrhea			
Drug allergy			
Fatigue			
Myalgia			
Arthralgia			
Fever			
Dizziness			
Hypocalcemia			
Others			
