# Supplementary material for: Randomized clinical trial comparing efficacy and safety of brand versus generic alendronate (Bonmax®) for osteoporosis treatment
Source: PLoS One. 2017 Jul 5;12(7):e0180325. doi: 10.1371/journal.pone.0180325 (PMC5498028; doi:10.1371/journal.pone.0180325)
Supplement: S2 File — (PDF) [file pone.0180325.s002.pdf]

Study protocol

Randomized trial comparing efficacy and safety of brand versus generic alendronate for osteoporosis treatment.

Aasis Unnanuntana, MD, MSc
Atthakorn Jarusriwanna, MD
Panupan Songcharoen, MD

Department of Orthopaedic Surgery,
Faculty of Medicine Siriraj Hospital,
Mahidol University,
Bangkok, Thailand

Contents

Protocol identification and investigator **3**
1. Title 3
2. Principle investigator 3
3. Co-investigators 3
4. Research funding 3
5. Location of clinical study 4
6. Duration of clinical study 4
7. This clinical study is a part of graduation or independent study 4
8. Study protocol 4
   - 8.1 Background/Rationale 4
   - 8.2 Objective 5
   - 8.3 Research type 6
   - 8.4 Research design 7
   - 8.5 Research subjects 7
   - 8.6 Research process 9
   - 8.7 Data collection process 9
   - 8.8 Outcome measurement/Data analysis 10
   - 8.9 References 10

Ethical consideration **9**
9. Characteristics of participants 12
10. Biological products or specimen collection/storage 12
11. Recruitment process 12
    - 11.1 Location 12
    - 11.2 Process 12
12. Informed consent process 12
13. Benefit from the clinical study 13
14. Adverse events 13
15. The effects of this clinical study to the religious belief or local traditions 13
16. The protection of the participant data 13

Case record form **14**

Protocol identification and investigator

1. Title

Randomized trial comparing efficacy and safety of brand versus generic alendronate for osteoporosis treatment

2. Principle investigator

Aasis Unnanuntana, MD, MSc
Associate Professor and Chief of Metabolic Bone Diseases Division
Department of Orthopaedic Surgery, Faculty of Medicine Siriraj Hospital,
Mahidol University, Bangkok, Thailand
Tel. +66-2419-7968-9
Email address: uaasis@gmail.com, siaun@mahidol.ac.th

3. Co-investigators

Atthakorn Jarusriwanna, MD
Resident
Department of Orthopaedic Surgery, Faculty of Medicine Siriraj Hospital,
Mahidol University, Bangkok, Thailand
Tel. +66-2419-7968-9
Email address: wonton2ton@hotmail.com

Panupan Songcharoen, MD
Professor
Department of Orthopaedic Surgery, Faculty of Medicine Siriraj Hospital,
Mahidol University, Bangkok, Thailand
Tel. +66-2419-7968-9
Email address: panupan.son@mahidol.ac.th

4. Research funding

❑ No

☑ Yes, please identify: this study was partially supported by the Medical Association of Thailand (Prasert Prasarttong-Osoth) Research Fund

5. **Location of clinical study**

☑ Single center, please identify: Siriraj Hospital, Faculty of Medicine Siriraj Hospital, Mahidol University, Bangkok, Thailand

❏ Multiple centers, please identify:

6. **Duration of clinical study**

Total duration: 3 years and 2 months

Data collection: 2 years and 6 months

Activity	Duration
Preparation, Institutional review board (IRB) submission, and funding	1 February 2014 – 30 April 2014
Data collection	1 May 2014 – 31 October 2016
Data analyses	1 November 2016 – 31 December 2016
Manuscript preparation and submission	1 January 2017 – 31 March 2017

7. **This clinical study is a part of graduation or independent study**

☑ No

❏ Yes, please identify:

8. **Study protocol**

8.1 Background/Rationale

Osteoporosis is a systemic disease characterized by compromised bone strength, predisposing to an increased risk of fracture.[1-5] Fragility fracture, defined as fracture after fall from a standing height or less, are associated with premature mortality and substantial decrements in quality of life.[1-3,6] In addition, both short- and long-term care for patients with fragility fractures place an enormous economic burden on health care system.[4,7,8] Due to high personal and societal costs of fragility fracture, their prevention is critically important. Once an individual has been found to carry a high risk of fracture such as those who diagnosed with osteoporosis or those with history of fragility fractures, an appropriate pharmacological intervention should be employed.

Based on the 2010 Thai osteoporosis guidelines, bisphosphonate is considered as a first line therapy.[5,9] Bisphosphonates have been shown to significantly reduce the risk of both vertebral and non-vertebral including hip fractures by reducing bone turnover, increasing bone mass and thus improving bone strength.[5,9,10] Alendronate is one of the most commonly used oral bisphosphonates, which is available in a once-weekly formulation and is approved for the treatment and prevention of postmenopausal osteoporosis. Previous study showed continued efficacy and safety of alendronate out to ten years of administration.[10]

Because of an escalating cost of health care, government in many countries have applied numerous measures to minimize expenditures. One of the most commonly used cost-saving method is to encourage physicians to use generic substitution for brand drugs after the patent of brand drugs has expired. Since alendronate is the first commercially-marketed amino-bisphosphonate for the treatment of osteoporosis, it is also the first amino-bisphosphonate to lose its patent and be provided to the market as a generic drug. Generally, insurances and health care providers prefer physicians to prescribe generic alendronate instead of brand drug, due to its lower costs. Although it is expected that generic alendronate will have the same clinical efficacy as the brand formulation based on the bioequivalence data, clinical information on bone mineral density (BMD), fracture reduction and side effects with new generic alendronate is limited.

8.2 Objective

8.2.1 Primary outcome

To evaluate the efficacy of a new generic alendronate (Bonmax®) comparing to brand alendronate (Fosamax®). The efficacy of generic alendronate will be determined by measuring the percent changes of bone mineral density at lumbar spine after 1 year of treatment and then comparing to those changes in the brand alendronate group.

8.2.2 Secondary outcome

1) Evaluate the efficacy of generic alendronate comparing to brand alendronate by measuring the percent changes of bone mineral densities at femoral neck and total hip from baseline to 1-year after treatment.

2) Evaluate the percent changes of serum bone markers (β-CTX and P1NP) from baseline to 3-months, 6-months, and 1-year after treatment between generic and brand alendronate group.
3) Evaluate the safety of generic alendronate comparing to brand alendronate at 3-months, 6-months, and 1-year after treatment by occurrence of the adverse events in the participants.
4) Evaluate the quality of life by EQ-5D™ questionnaire at 1-year after treatment between generic and brand alendronate group.

8.3 Research type

☑ Experimental biomedical/Clinical research

❑ Drug trial, please identify:

❑ Registered drug

❑ Investigational (new) drug

❑ Medical device trial, please identify:

❑ Registered device

❑ Investigational (new) device

❑ Vaccine trial, please identify:

❑ Registered vaccine

❑ Investigational (new) vaccine

☑ Experimental procedure/intervention, please identify: treatment with generic alendronate (Bonmax®) or brand alendronate (Fosamax®)

❑ High risk ☑ Minimal risk

❑ Bioequivalence

❑ In vitro/laboratory-based study

❑ Research using repository of biological products (cells, blood, tissues, fluids, etc.)

❑ Others, please identify:

❑ Observation clinical research

❑ Prospective (cohort) study

❑ Case series

❑ Retrospective (chart) review

❑ Epidemiology research

❑ Surveillance

❑ Monitoring

❑ Others, please identify:

❑ Social/Behavioral research

❑ Questionnaire-based research

❑ Others, please identify:

8.4 Research design

☑ Randomized-controlled trial

❑ Quasi-experimental study (manipulation and control only, without randomization)

❑ Pre-experimental study (manipulation only, without control and randomization)

❑ Prospective cohort study

❑ Descriptive study

❑ Cross-sectional study

❑ Pilot study

❑ Others, please identify:

8.5 Research subjects

Sample size calculation

The sample size calculation formula for comparison the 2-mean independent population using in this study is shown below.[11,12]

$$n = \frac{(Z_\alpha + Z_\beta)^2(\sigma_0^2 + \sigma_1^2)}{\delta^2}$$

In order to test non-inferiority between generic and brand alendronate groups, the standard deviation of the BMD change at the lumbar spine from baseline to 1-year post-treatment of the brand alendronate (Fosamax®) is used. A previous investigation[13] (Grima DT, 2010) found that the standard deviation of the BMD at the lumbar spine in patients who received brand alendronate was 0.138. Based on the results of that study, a non-inferiority margin between brand and generic alendronate

was set equal to half of the standard deviation of the brand alendronate group. Power analysis and sample size calculations indicated that a sample size of 50 patients per group would provide 80% statistical power to detect this effect size between the two groups (alpha = 0.05, beta = 0.20). Since recruitment was increased by 20% to allow for loss to follow-up and poor compliance, a total of 60 patients per group were required for this study.

Inclusion criteria

Patients who are postmenopausal women or men who aged older than 50 years and meet the indications for osteoporosis treatment according to the Thai Osteoporosis Foundation's 2010 treatment guidelines.

1) History of spinal or hip fractures with low energy trauma.
2) BMD by Dual energy X-ray absorptiometry (DXA) scan with T-score ≤ -2.5 at the femoral neck, total hip, or L1-L4 spine.
3) BMD by DXA scan with T-score between -1 and -2.5 at the femoral neck, total hip, or L1-L4 spine and a 10-year hip fracture probability ≥ 3% or a 10-year major osteoporosis-related fracture probability ≥ 20% based on Fracture risk assessment tool. (FRAX™)

Exclusion criteria

1) Patients who have contraindications to use bisphosphonates e.g. gastroesophageal reflux disease or drug allergy to bisphosphonates.
2) Patients with an abnormality of serum calcium levels. (more than 10.2 mg/dl or less than 8.5 mg/dl)
3) Patients with estimated glomerular filtration rate less than 35 mL/min/1.73 m^2
4) Patients with metabolic bone diseases such as hyperparathyroidism, Paget's disease, renal osteodystrophy, rheumatoid arthritis, severe vitamin D deficiency (serum 25-hydroxyvitamin D < 10 ng/mL), etc.
5) Patients who were received anti-osteoporotic drugs during the past 1 year.
6) Patients who currently taking steroids ≥ 5 mg/day within 6 months.

Withdrawal or termination criteria

1) Patients with severe adverse effects or allergy to drugs or suffer from a new major fracture that surgeon concerns of potential effects of bisphosphonate on fracture healing process.
2) Participants who decline for participation.
3) The interim analysis is found significant inferiority or harm to the patients after treatment with generic alendronate comparing to brand alendronate.

8.6 Research process

After signing the consent, patients will be allocated into 2 groups: generic alendronate (Bonmax®) and brand alendronate (Fosamax®). All patients will be given calcium orally 1,000 mg/day, proper oral vitamin D dosage to maintain normal vitamin D level, and oral bisphosphonates weekly either with generic or brand alendronate for approximately 1 year. Bone mineral density and bone markers (serum β-CTX and serum P1NP) will be measured in each patient. Bone mineral density will be performed at baseline and after 1-year of treatment while bone markers will be sent at baseline, 3, 6 and 12 months after treatment. Percent changes of bone mineral density from baseline to 1-year after treatment will then be compared between generic and brand alendronate. Furthermore, the percent changes of serum bone markers from baseline to 1-year after treatment will also be compared between the 2 groups. In addition, the occurrence of adverse events associated with bisphosphonate use in each group will be collected.

8.7 Data collection process

Data of the participants were collected in case record form. (Please see the case record form in the appendix.)

1) Date of enrollment
2) Patient demographic data such as gender, age, hometown, educational status, body weight, height, underlying diseases, current medications
3) Indication for osteoporosis treatment
4) Previous surgical history
5) Previous fracture history
6) Laboratory investigation: hemoglobin (Hb), hematocrit (Hct), BUN, creatinine (Cr), AST, ALT, alkaline phosphatase (ALP), vitamin D level

[25(OH)D], total calcium, phosphate, parathyroid hormone (PTH), bone markers (β-CTX and P1NP)

7) Bone mineral density measurement
8) Adverse effects such as nausea, vomiting, heartburn, abdominal bloating, constipation, diarrhea, urticarial, myalgia, others
9) Quality of life questionnaire (EQ-5D™ Thai version). The EQ-5D™ Thai version is designed for self-completion by the participant and contains two parts: the EQ-5D-5L utility score (EQ-US) and the EQ visual analogue scale (EQ-VAS).[14] Here, the authors used the EQ-VAS. Patients were asked to score their health status on a visual analog scale (VAS) that ranged from 0 to 100. The top score of the scale (100) represents the best imaginable health state while the bottom (0) represents the worst imaginable state.

8.8 Outcome measurement/Data analysis

Data are presented as number, percentages (%) for categorical variables, or mean ± standard deviation for continuous variables. Baseline patient characteristics and the results of both groups were assessed for normality with the Kolmogorov–Smirnov test and were compared using Pearson's chi-square or Fisher's exact test for categorical variables. As for continuous variables, a Student's t-test was used to compare parametric data, and a Mann–Whitney U test was used to compare nonparametric data. The comparison of the percentage of patients with gain, loss, and stable BMDs at the lumbar spine, total hip, and femoral neck was analyzed by a chi-square test. One-way repeated measures ANOVA was used to assess the effect of time on the change in each bone turnover marker of each patient group. All analyses were performed using SPSS version 18.0 (SPSS, Inc., Chicago, IL, USA). A p-value < 0.05 was regarded as statistically significant.

8.9 References

1) Brown JP, Davison KS, Olszynski WP, Beattie KA, Adachi JD. A critical review of brand and generic alendronate for the treatment of osteoporosis. SpringerPlus 2013;2:550.
2) Ringe JD, Möller G. Differences in persistence, safety and efficacy of generic and original branded once weekly bisphosphonates in patients with postmenopausal

osteoporosis: 1-year results of a retrospective patient chart review analysis. Rheumatol Int 2009;30:213-221.

3) Kanis JA, Reginster JY, Kaufman JM, Ringe JD, Adachi JD, Hiligsmann M, Rizzoli R, Cooper C. Reappraisal of generic bisphosphonates in osteoporosis. Osteoporos Int 2012;23:213-221.
4) Pongchaiyakul C, Songpattanasilp T, Taechakraichana N. Burden of osteoporosis in Thailand. J Med Assoc Thai 2008;91(2):261-267.
5) National Osteoporosis Foundation. Clinician’s guide to prevention and treatment of osteoporosis. Washington, DC: National Osteoporosis Foundation; 2013.
6) van den Bergh JPW, Bouts ME, van der Veer E, van der Velde RY, Janssen MJW, Geusens PP, Winkens B, Oldenhof NJJ, van Geel TACM. Comparing tolerability and efficacy of generic versus brand alendronate: A randomized clinical study in postmenopausal women with a recent fracture. Plos One 2013;8:10.
7) Reginster JV, Burlet N. Osteoporosis: A still increasing prevalence. Bone 2006;38:S4-S9.
8) Burge R, Dawson-Hughes B, Solomon DH, Wong JB, King A, Tosteson A. Incidence and economic burden of osteoporosis-Related fractures in the United States, 2005-2025. J Bone Miner Res 2007;22(3):465-475.
9) Royal College of Orthopaedic Surgeons of Thailand. Clinical practice guideline in osteoporosis treatment. Bangkok: Royal College of Orthopaedic Surgeons of Thailand; 2010.
10) Black DM, Cummings SR, Karpf DB, Cauley JA, Thompson DE, Nevitt MC, Bauer DC, Genant HK, Haskell WL, Marcus R, Ott SM, Torner JC, Quandt SA, Reiss TF, Ensrud KE. Randomised trial of effect of alendronate on risk of fracture in women with existing vertebral fractures. Lancet 1996;348:1535-1541.
11) Chow SC, Shao J, Wang H. Sample size calculations in clinical research. Basel: Marcel Dekker, Inc.;2003.
12) Hayes RJ, Benett S. Simple sample size calculation for cluster-randomized trials. Int J Epidemiol 1999;28:319-326.
13) Grima DT, Papaioannou A, Airia P, Ioannidis G, Adachi JD. Adverse events, bone mineral density and discontinuation associated with generic alendronate among postmenopausal women previously tolerant of brand alendronate: a retrospective cohort study. BMC Musculoskelet Disord 2010;11:68.

14) Kimman M, Vathesatogkit P, Woodward M, E ST, Thumboo J, Sukit Yamwong S, Ratanachaiwong W, Hwee LW, Sritara P. Validity of the Thai EQ-5D in an occupational population in Thailand. Qual Life Res 2013;22:1499–1506.

Ethical consideration

9. Characteristics of participants

❑ Healthy volunteers

☑ Patients excluding vulnerable subjects

❑ Others e.g. Retrospective chart review

❑ Vulnerable subjects, please identify:

10. Biological products or specimen collection/storage

☑ No

❑ Yes, please identify:

11. Recruitment process

11.1 Location

The Metabolic Bone Diseases (MBD) and Osteoporosis Clinic, Siriraj Hospital, Faculty of Medicine Siriraj Hospital, Mahidol University, Bangkok, Thailand

11.2 Process

The co-investigators and research assistant explain the details and characteristics of the study protocol and enrollment to the patients who met all the study entry criteria.

12. Informed consent process

❑ Not applicable due to retrospective chart review

❑ Waive for inform consent

☑ Need to apply for inform consent

The patients will sign the consent form if they accept the study protocol after explanation by the co-investigators and research assistant.

13. Benefit from the clinical study

The benefit of this study is clear. If adequate efficacy of generic alendronate could be established and if it affords the same safety profile as those of brand alendronate, the use of generic alendronate could then be recommended. The patients will be prescribed with an anti-osteoporosis drug with a more affordable price which may help reducing the healthcare expenditures.

14. Adverse events

The possible side effects and adverse events are stomachache or gastrointestinal reflux, nausea and vomiting, abdominal bloating, diarrhea, fatigue with myalgia or arthralgia, fever, dizziness, hypocalcemia, osteonecrosis of jaw, and drug allergy.

15. The effects of this clinical study to the religious belief or local traditions

❑ Yes, please describe how to solve the problem:

☑ Not applicable

16. The protection of the participant data

❑ This study doesn't record any private data of the participants

☑ This study does record the private data of the participants

If any private data of the participants are recorded, the investigators need to demonstrate how the data is protected: all data were stored and saved in the electronic files in a password-protected computer for at least 5 years after the study completion.

Case no.______________

Date of enrollment______________

Case record form

Demographic data

Gender ❑ Male ❑ Female

*Age*_________________ years old

Hometown ❑ Bangkok and vicinity ❑ Northern ❑ North-eastern

❑ Central ❑ Eastern ❑ Southern

Occupation ❑ None/Housewives ❑ Government officers ❑ Agriculture/Farmers

❑ Private company officers ❑ Students ❑ Others________________________

Educational status ❑ None ❑ Primary school

❑ High school ❑ University/College

*Body weight*___________________ kg *Height*____________________ cm

*BMI*___________________ kg/m^2

Underlying diseases ❑ No

❑ DM ❑ HT ❑ Dyslipidemia ❑ CKD ❑ Liver diseases

❑ Asthma/COPD ❑ Heart diseases/CAD ❑ Stroke/CVA

❑ Others____

*Current medications*____

____

Case no.______________

Previous surgical history ❑ Yes____ ❑ No

Previous fracture history ❑ Yes____ ❑ No

Indication for osteoporosis treatment: Postmenopausal women or men age 50 and older with

❑ History of spinal or hip fractures with low energy trauma

❑ BMD by DXA scan with T-score ≤ -2.5 at the femoral neck, total hip or L1-L4 spine

❑ BMD by DXA scan with T-score between -1 and -2.5 at the femoral neck, total hip or L1-L4 spine and a 10-year hip fracture probability ≥ 3% or a 10-year major osteoporosis-related fracture probability ≥ 20% based on FRAX™

Laboratory and clinical results

Time / Lab		Baseline Date___________	3rd month Date___________	6th month Date___________	12th month Date___________
Hb/Hct					
BUN/Cr					
GFR					
Calcium					
Phosphate					
AST					
ALT					
ALP					
PTH					
25(OH)D					
CTX					
P1NP					
BMD	L1-L4				
	Femoral neck				
	Total hip				
EQ-5D™					

Case no.______________

Complications/Side effects

Complications	3rd month<br>Date_____________	6th month<br>Date_____________	12th month<br>Date_____________
Stomachache/reflux			
Nausea/vomiting			
Bloating			
Diarrhea			
Drug allergy			
Fatigue			
Myalgia			
Arthralgia			
Fever			
Dizziness			
Hypocalcemia			
Others			
